# Peptide-Based Subunit Vaccine Design of T- and B-Cells Multi-Epitopes against Zika Virus Using Immunoinformatics Approaches

**DOI:** 10.3390/microorganisms7080226

**Published:** 2019-07-31

**Authors:** Vivitri Dewi Prasasty, Karel Grazzolie, Rosmalena Rosmalena, Fatmawaty Yazid, Fransiskus Xaverius Ivan, Ernawati Sinaga

**Affiliations:** 1 Faculty of Biotechnology, Atma Jaya Catholic University of Indonesia, Jakarta 12930, Indonesia; 2 Department of Biology, Faculty of Life Science, Surya University, Tangerang, Banten 15143, Indonesia; 3 Department of Medical Chemistry, Faculty of Medicine, Universitas Indonesia, Depok 16424, Indonesia; 4 Faculty of Biology, Universitas Nasional, Jakarta 12520, Indonesia

**Keywords:** Zika virus, peptide vaccine, epitope, immunoinformatics, molecular docking, molecular dynamics

## Abstract

The Zika virus disease, also known as Zika fever is an arboviral disease that became epidemic in the Pacific Islands and had spread to 18 territories of the Americas in 2016. Zika virus disease has been linked to several health problems such as microcephaly and the Guillain–Barré syndrome, but to date, there has been no vaccine available for Zika. Problems related to the development of a vaccine include the vaccination target, which covers pregnant women and children, and the antibody dependent enhancement (ADE), which can be caused by non-neutralizing antibodies. The peptide vaccine was chosen as a focus of this study as a safer platform to develop the Zika vaccine. In this study, a collection of Zika proteomes was used to find the best candidates for T- and B-cell epitopes using the immunoinformatics approach. The most promising T-cell epitopes were mapped using the selected human leukocyte antigen (HLA) alleles, and further molecular docking and dynamics studies showed a good peptide-HLA interaction for the best major histocompatibility complex-II (MHC-II) epitope. The most promising B-cell epitopes include four linear peptides predicted to be cross-reactive with T-cells, and conformational epitopes from two proteins accessible by antibodies in their native biological assembly. It is believed that the use of immunoinformatics methods is a promising strategy against the Zika viral infection in designing an efficacious multiepitope vaccine.

## 1. Introduction

Zika Virus is an arbovirus of the genus Flavivirus known for causing Zika disease/fever in humans. Zika virus originates from Africa [1,2], and it has been around for a long time in Africa and South Asia [3,4]. Since human infections only happened sporadically with minimal to no symptoms, Zika remained largely overlooked until the 2007 outbreak at Yap Island in the Pacific Ocean [3]. It then continued to spread eastward while accumulating mutations that gave rise to some serious health problems, including microcephaly and the Guillain–Barré syndrome [1,4,5,6,7]. In late 2014, Zika entered America through Brazil, and promptly caused a major outbreak, reaching many countries in South and Central America, the Caribbean and several states within the United States [3,4,8,9]. In 2016, there were 205,578 probable Zika cases in Brazil [10]. From January 2015 to November 2016, there were 304 cases of microcephaly with a confirmed link to the Zika infection [11]. 

Although Zika is a potentially serious disease, there has been no medication or vaccine for Zika. Current treatments like rest, administration of fluids, and analgesics [3,4] only deal with the resulting symptoms. To directly target the virus, several Zika vaccines are currently under development, using various parts of the virus as their basis [12]. Some of them have reached clinical or preclinical trial stages, with one DNA-based vaccine currently in its second phase clinical trial [12]. Safety becomes the main concern in creating a Zika vaccine, considering pregnant women and infants are in the vulnerable population and at risk of antibody-dependent enhancement (ADE) upon entry of a related Flavivirus. ADE is the immunogenic mechanism for more severe virus infection, for example, secondary Dengue virus infections. Antibodies targeted for the virus envelope proteins are supposed to neutralize the virion by blocking it from binding to the receptors of fragment-crystallizable (Fc) on the surface of certain cells. If the affinity of the antibody from a primary infection is too low for a different serotype of the virus, the virion is not neutralized, and the secondary infection is increased [13]. ADE might arise when the production of reactive, non-neutralizing antibodies help the propagation of a related virus strain by facilitating antigen entry [14], and it has been observed in the dengue infection [15].

The peptide vaccine is seen as a safer platform for vaccine development. By only using the parts of a protein that can elicit an immune response, unnecessary components that are potentially antigenic can be eliminated. In the case of Zika, the CD8+ T-cell activity, a target of this vaccine, has been shown to play a protective role against ADE in the dengue infection [16,17]. Immunoinformatics approaches have been proven suitable for accurately determining 18 peptides from the ZIKV envelope containing predicted HLA-I T-cell epitopes and investigated T-cell cross-reactivity between ZIKV-infected individuals and DENV-vaccinated subjects by IFNγ ELISPOT [18]. Several in silico studies have searched epitopes on Zika, but most tended to focus on finding CD8+ T-cell epitopes, with a limited amount of further analyses like molecular docking and molecular dynamics [16,19,20,21,22,23,24]. A part of the reason for the popularity of CD8+ T-cells as a target is the more developed MHC-I epitope prediction methods [25]. The overall design of this study was to use the Zika protein sequence data to obtain candidate epitopes for CD4+ T-cell, CD8+ T-cell, and B-cell (linear epitopes), and also Zika 3D protein structures to find conformational B-cell epitope candidates in silico. The findings were verified by molecular docking and molecular dynamics analyses.

## 2. Materials and Methods

### 2.1. Preparation of Zika Polyprotein Sequences

Polyprotein sequences for Zika viruses were obtained from the NCBI database (http://www.ncbi.nlm.nih.gov/). The 39 American sequences were obtained from the BioProject accession: PRJNA344504 [8]. 

### 2.2. Preparation and Visualization of Three-Dimensional Protein Structures

Three-dimensional (3D) protein structures with ID codes were required for Zika proteins and HLA molecules (PDB ID for MHC-I receptors: 2GIT, 2X4O, 5TXS, 3LKN, 1QQD; and MHC-II receptors: 1UVQ, 4MD5, 1A6A, 1BX2, 5V4M) were obtained from the Protein Databank (PDB) (http://www.rcsb.org/pdb/). In addition, some of these proteins would not be readily available and require modeling. For proteins to be modeled, a 3D structure of a template protein was downloaded instead. Template-based protein structures were decided based on their correlation to the target protein, sequence similarity, and model quality. Visualization of protein structures was done by the software DS Visualizer [26] and PyMol [27].

### 2.3. CD8+ T-cell Epitope Prediction

The protein sequence in the FASTA format was used as input for the prediction tools. The prediction was done using NetCTLpan1.1 [28] which uses the ANN algorithm used in NetMHCpan [29], combined with predictions of proteasomal cleavage from the NetChop [30] and TAP transport efficiency [31]. For validation, MHC-I Binding Prediction tools from the Immune Epitope Database (IEDB) (http://tools.iedb.org/mhci/) were employed by using the recommended method. The recommended method combines prediction results from several prediction methods into one consensus result when possible [32]. Prediction methods used from IEDB’s collection were NetMHCpan 3.0 [29,33], NetMHC 4.0 [34,35], SMM [36] and Combinatorial Library [37]. 

### 2.4. CD4+ T-cell Epitope Prediction

The protein sequence in the FASTA format was used as input for the prediction tools. The prediction was done at IEDB (http://tools.iedb.org/mhcii/) by using the recommended method. The IEDB recommended it combines the results from several prediction methods to obtain one consensus result when possible [38]. Prediction methods used from IEDB’s collection were the Combinatorial Library [37], SMM-align [39], NN-align [40], Sturniolo [41] and NetMHCIIpan 3.1 [42]. Validation was done using PREDIVAC [43] for HLA-DR epitopes and NetMHCIIpan 3.2 [44] for HLA-DQ and DP epitopes. PREDIVAC uses the concept of specificity determining residues in predicting peptide binding [43]. 

### 2.5. T-cell Epitope Shortlisting

The predicted T-cell epitopes were then subjected to a series of analyses. Analysis of the pMHC-I- T-cell receptor (TCR) binding was done for MHC-I epitopes using the IEDB Class I Immunogenicity Predictor (http://tools.iedb.org/immunogenicity/) [45], where non-immunogenic epitopes (negative-scoring) were eliminated. The peptide toxicity analysis was done using ToxinPred [46]. Following that, the epitopes were compared against an expanded pool of Zika polyprotein sequences, consisting of 39 initial sequences and 15 new sequences (Table S1, Supplementary file) from South East Asia, French Polynesia and Mexico, to check for their level of conservation at their respective positions across the sequences. 

The population coverage analysis was then done on conserved epitopes. A list of HLA alleles that would bind to each epitope was gathered. The program NetMHC 4.0 [34,35] was used for identifying MHC-I epitopes, and NetMHCII 2.3 [44] was used for identifying MHC-II epitopes. Epitopes were tested for all available HLA-A, B, C, DR, DP, and DQ alleles. The epitope: HLA pair list was then used to estimate population coverages for nine regions: North America, Central America, South America, South Asia, South East Asia, West Indies, Oceania, Central Africa, and West Africa by using the tool provided by IEDB (http://tools.iedb.org/population/) [47].

Following the population coverage analysis, ten best epitopes for each HLA class were gathered. A cutoff of 80% averaged coverage was used, then the numbers of strong (SB) and weak binders (WB) were considered. To give higher weight on strong binders, WB was given half the value of an SB. Peptide antigenicity was checked by comparing each epitope to the complete sequence that ran through the EMBOSS antigenic [48] and looking for the presence of antigenic residues. Lastly, to prevent autoimmune responses, BLASTp [49] was performed for each epitope against human proteome at default settings. Epitopes with 100% sequence similarity, one indel or one mutation from the corresponding human sequence were eliminated. 

### 2.6. Linear and Conformational B-cell Epitope Prediction

Prediction for linear epitopes was done using BepiPred-2.0 [50] and LBtope [51]. BepiPred-2.0 was trained using the Random Forest algorithm to find epitope residues from a dataset of epitopes and non-epitopes in crystal structures [50], and LBtope was trained using various methods including the SVM and Nearest Neighbor on various datasets [51]. The dataset LBtope_confirm was used in the epitope prediction, all entries in this dataset had been reported by at least two studies [51]. For BepiPred-2.0, residues scoring above ~0.5 were considered epitope, and for Lbtope, residues with %probability above ~60 were considered epitope. The minimal length of a linear epitope was set to be six, following the typical minimum epitope length of Zika epitopes listed in IEDB (http://www.iedb.org) [52].

To predict conformational epitopes, three-dimensional Zika protein structures were obtained either directly from PDB or through modeling. The prediction was done using DiscoTope 2.0 [53] by uploading the protein PDB file and specifying the chain(s) to be used. DiscoTope 2.0 combines sequence information with spatial information in finding epitope residues [53]. 

### 2.7. B-cell Epitope Shortlisting

Similar to the T-cell, consensus epitopes were passed through toxicity, conservancy, and antigenicity tests, then their similarity to human proteins was checked using BLASTp [49]. In similarity checking, epitopes with identity above 90% with a part of human proteome were eliminated.

### 2.8. Modeling of Protein Structures

All modeling was done using MODELLER 9.19 [54]. For the homology modeling, template proteins were first obtained by the help of “build_profile()” command or searching in PDB. After obtaining a template structure, the target sequence was aligned to the template using the command “salign()”, and the model was built using “automodel()”. Generated 3D structures were then validated using Errat [55], ProQ [56], Verify3D [57] and MolProbity [58].

Modeling for proteins with minimal (1aa) difference from the target sequence was done by changing only the residue of interest while leaving the other residues rigid. MODELLER would then optimize the new residue. 

### 2.9. Molecular Docking Simulation

PDB structures of both the epitope (ligand) and MHC were used in docking. Epitope 3D structures were generated using PEP-FOLD3 [59] by inputting their amino acid sequence. AutoDock Vina 1.1.2 [60] was employed to perform docking simulations, with the inputs of both PDB files and docking parameters.

Docking preparation was done in AutoDock Tools [61]. Firstly, protein orientation was adjusted such that minimum xyz dimensions for the peptide binding site could be achieved. Protein was given polar hydrogens, and ligand torsions were enabled for all rotatable bonds, except for the amide bonds. After that, a grid box was set to be large enough to accommodate the peptide ligand (at least 30 Å x 15 Å x 15 Å in size). Docking was done using default parameters, save for exhaustiveness = 16. 

### 2.10. Molecular Dynamics Simulation

A molecular dynamics simulation was performed to study the stability of docked peptide-MHC complexes. Docked epitopes were complexed with their HLA receptors and became input for MD simulation performed by GROMACS 2018 [62,63]. 

The force field AMBER99sb-ILDN [64] was used with the TIP3P water model [65]. The simulation condition was 310 K, 1 bar, and 0.15 M NaCl to mimic the physiological condition. The simulation box was a dodecahedron with an additional 1.2 nm space from the edge of the protein. The atom neighbor list was searched using the Verlet cutoff method [66], and electrostatic forces were calculated using the smooth PME method [67] with a cutoff distance of 1 nm. Minimization was done using the steepest descent down to 1000 kJ/mol.nm. Bond lengths for all atoms were constrained using the LINCS algorithm [68]. Equilibration was done by restraining heavy atom (non-H) positions. Temperature equilibration was done using the v-rescale thermostat [69] for 100 ps, followed by the pressure equilibration for 500 ps using the Berendsen thermostat (2 fs timestep). Finally, position restraints were lifted; barostat was switched to Parrinello–Rahman [70,71]; and the production simulation was run for 20 ns (2 fs timestep).

## 3. Results

### 3.1. Preparation of Zika Polyprotein Sequence for Epitope Screening

Metsky et al. [9] uploaded 110 genome sequences of circulating Zika viruses in the Americas (American’s Zika lineage) alongside their translated protein sequences, under the BioProject accession PRJNA344504. A sequence pool was created from 39 polyprotein sequences that contained no long stretches of gap (Table S1, Supplementary file). These sequences came from six countries/states: Dominican Republic, Colombia, Florida, Puerto Rico, Brazil, and Honduras. From the sequence pool, one consensus sequence was generated by preferring Brazilian sequences, since the strains circulating in Brazil are the most established. Sporadic mutations (occurring random or in scattered mutations) were reverted to their corresponding consensus residues as new sequences, and localized mutations (regular mutation) were reverted to their corresponding Brazilian sequences. This sequence is 3242 amino acid long and available in Diagram S1 (Supplementary file). 

### 3.2. Selection of HLA Alleles in T-cell Epitope Screening

The HLA alleles used in this study are summarized in Table 1. In total, a pool of 13 HLA class I alleles for HLA-A and B was created. Out of the 13 alleles, nine alleles have been commonly found in Indonesia and four alleles have been reported to have a protective effect against dengue. For class II, a pool of 12 alleles for HLA-DR and DQ was created, consisting of 10 common alleles and three protective alleles. In cases where the allele subtype was not known, 01 was used to represent that allele. To form the binding pocket of HLA class II molecules, chain A and B need to be combined. However, due to the monomorphic nature of HLA-DRA [72], the recombination of chain A and B was only done for the five HLA-DQ alleles, resulting in a set of 13 class II HLA molecules. 

### 3.3. Prediction of T-cell Epitopes Recognized by MHC-I and MHC-II

Two predictors were used in both finding MHC-I epitopes (HLA-A and B) and MHC-II (HLA-DR and DQ) to generate consensus epitope-HLA pairs. Two hundred and seventy seven epitope-HLA pairs were discovered for MHC-I, and 95 pairs for MHC-II. By ignoring HLA, there were 175 unique peptides for class I and 67 for MHC-II. To ensure the epitopes would work well as a vaccine component, the quality of these epitopes was then further evaluated by running them through immunogenicity, toxicity, conservation and population coverage tests. Ten of the best epitopes for MHC-I and II each were obtained. After further antigenicity and autoimmunity testing, nine MHC-I epitopes (Table 2) and eight MHC-II epitopes (Table 3) were selected for molecular docking study.

### 3.4. Docking of T-cell Epitopes to MHC Molecules

Nine selected epitopes for MHC-I and eight selected epitopes for MHC-II were used for the molecular docking study. Each epitope was paired with an appropriate HLA molecule predicted to strongly bind the epitope. By looking at the NetMHC and NetMHCII results alongside the initial screening results, one HLA was selected to be paired with each epitope based on the binding quality, allele frequency in Southeast Asia, and prediction reliability (for NetMHCII). Some class II HLA structures were not available in PDB and had to be modeled. Structures for HLA-DQA1*05:01-DQB1*03:03, HLA-DQA1*02:01-DQB1*03:03 and HLA-DRA/DRB1*09:01 were generated using the homology modeling, while DRA/HLA-DRB1*15:02 that has only one residue difference from its reference structure was point mutated to its receptor. The PDB structures used to model these HLAs are listed in Table 4 and Table 5, whereas validation of the resulting structures can be seen in Table S2 (Supplementary file).

The AutoDock Vina uses its scoring function to calculate inter-atomic interactions and the difference of the Gibbs free energy (∆G) value, which depicts the binding energy between the protein and ligand [60,82]. This value can be converted into an inhibition constant (K_i_) by using the formula:

(1) $$K_{i}=\;e^{\left(\frac{\Delta G}{RT}\right)}$$

where R and T are the gas constant and temperature, respectively. A lower binding energy and inhibition constant indicate a stronger bond between the epitope and HLA.

**Figure 1:**
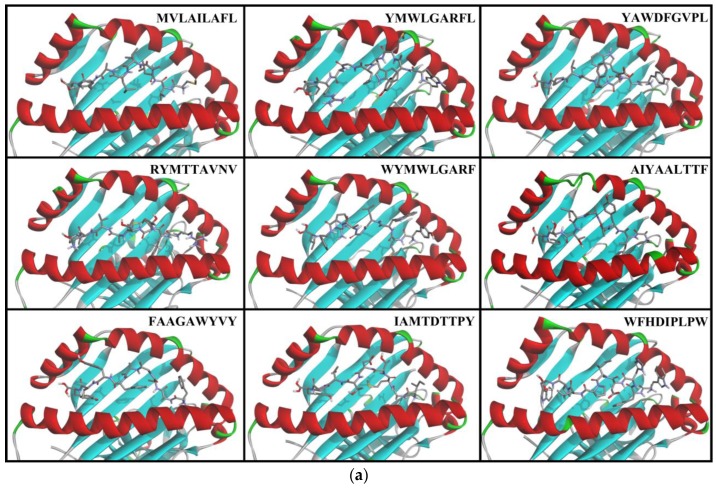

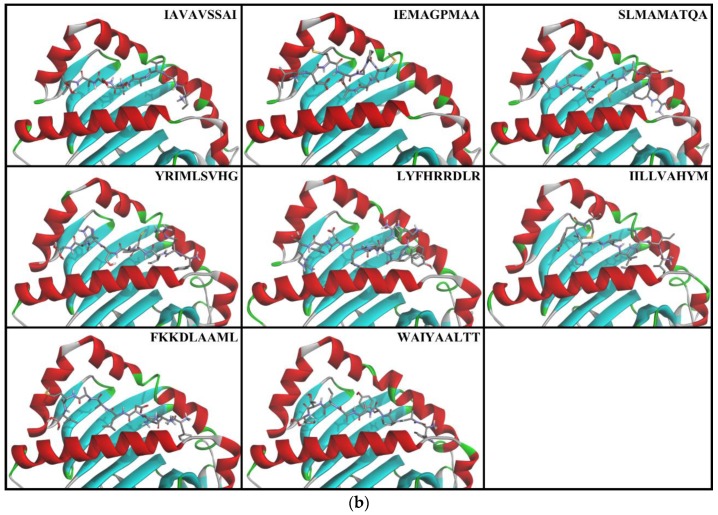
Conformations of epitopes when docked to a strongly binding HLA. (**a**) MHC-I epitopes; (**b**) MHC-II epitopes.

Peptide docked poses are displayed in Figure 1, and docking summaries are displayed in Table 4 and Table 5. The bolded entries WFHDIPLPW for MHC-I and WAIYAALTT for MHC-II had the lowest binding energies and inhibition constants (Ki) in their respective groups, indicating the best binding interactions.

### 3.5. Molecular Dynamics Simulations

**Figure 2:**
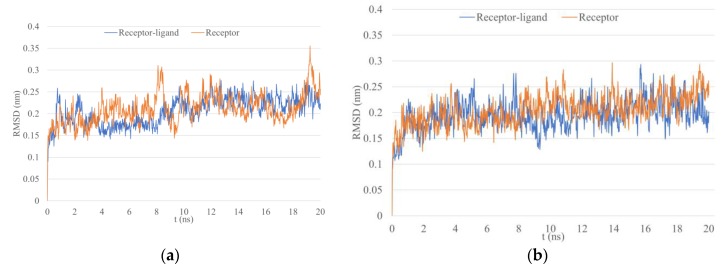
RMSD fluctuation within 20 ns MD simulation. (**a**) Epitope WFHDIPLPW with HLA-C*04:01; (**b**) epitope WAIYAALTT with HLA-DRB1*15:02.

The stability of HLAs and peptide epitopes were tested by 20 ns MD simulation. As a comparison, the same simulation was also done for the unbound form of the receptors. The RMSD analysis for WFHDIPLPW-HLA-C*04:01 (Figure 2a) showed that the stability was reached at about 10 ns for the complex structure, indicated by a plateauing RMSD value. The unbound HLA seemed to have not reached stability. The RMSD analysis for WAIYAALTT-HLA-DRA/DRB1*15:02 (Figure 2b) also showed a rather stable RMSD in the bound structure, whereas RMSD of the unbound structure appeared to increase slowly.

**Figure 3:**
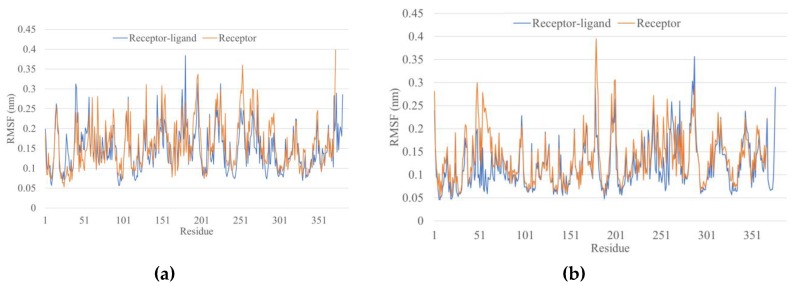
Residual RMSF after 20 ns simulation. (**a**) Epitope WFHDIPLPW with HLA-C*04:01; (**b**) epitope WAIYAALTT with HLA-DRB1*15:02.

**Figure 4:**
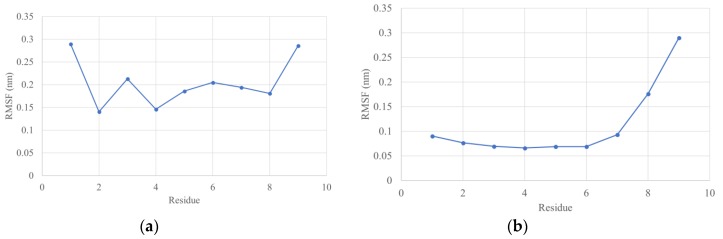
Residual RMSF after 20 ns simulation only for epitope residues. (**a**) WFHDIPLPW; (**b**) WAIYAALTT.

Root mean square fluctuation (RMSF) values depict the degree of movement of each residue. In both cases (WFHDIPLPW and WAIYAALTT), the overall RMSF appeared higher for the unbound structure, indicating a lower stability (Figure 3). The last nine residues in the complex RMSF belong to the epitope (Figure 4). WFHDIPLPW had a rather high RMSF (about 1.4-2.9 Å, average 2.04 Å), while WAIYAALTT had a lower RMSF (average 1.11 Å) with residues one to seven having RMSF lower than 1 Å (Figure 4). It could be inferred that residues one to seven of WAIYAALTT had been docked well, resulting in minimum changes (movements) and low RMSF values.

**Figure 5:**
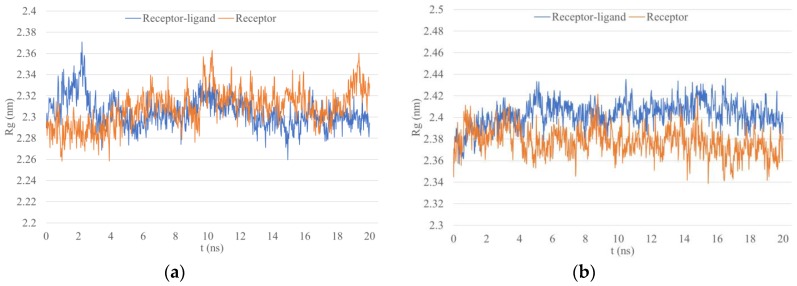
The radius of gyration (Rg) within 20 ns MD simulation. (**a**) Epitope WFHDIPLPW with HLA-C*04:01; (**b**) epitope WAIYAALTT with HLA-DRB1*15:02.

Lastly, the radius of gyration (Rg) analysis depicts structure density by calculating the atomic root mean square deviation (RMSD) from a center of mass [83]. A protein changing the conformation would thus display a changing Rg value. Rg values for WFHDIPLPW-HLA-C*04:01 tended to stabilize around 2.3 Å while in the unbound structure Rg seemed to yet stabilize after 20 ns (Figure 5). Rg values for WAIYAALTT-HLA-DRB1*05:02 both in the bound and unbound structure appeared rather stable with a slightly declining trend for the unbound structure. However, the unbound Rg was lower, implying a more compact structure (Figure 5). 

**Figure 6:**
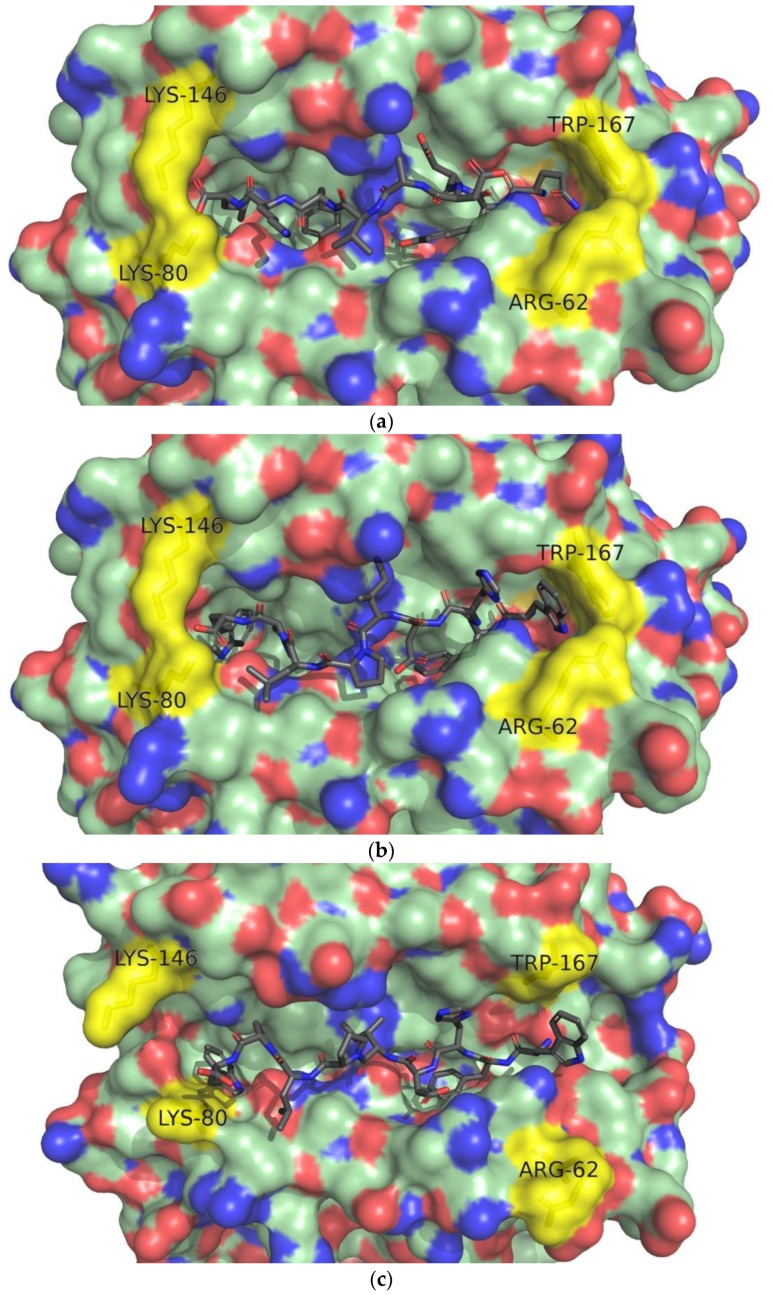
Conformational changes in MD simulation of WFHDIPLPW-HLA-C*04:01. (**a**) HLA-C*04:01 (PDB ID: 1QQD) bound with consensus epitope from pool sequencing (QYDDAVYKL); (**b**) epitope WFHDIPLPW docked to HLA-C*04:01; (**c**) epitope WFHDIPLPW bound to HLA-C*04:01 after 20 ns MD simulation. Epitope residues one to nine are numbered. Residues Arg62, Lys80, Lys146, Trp167 are labeled and colored yellow.

The conformational analysis revealed several changes in the bound ligand WFHDIPLPW after 20 ns of MD simulation (Figure 6). The docked structure shows residues two to three (FH), forming the kink in the ligand structure (Figure 6b). After 20 ns of MD simulation, the kink was observed in residues seven to nine (LPW) (Figure 6c). MD simulation also opened the ligand -COOH end part of the receptor (mainly Lys80 and Lys146) and the -NH_2_ end, which was closed by Arg62 and Trp167 [84] (Figure 6c).

Fleischmann et al. [85] reported that strongly binding epitopes tend to tighten the gap in the HLA molecule. According to the atoms used by Fleischmann et al. [85], d1 was defined as the distance between CA of Tyr85 and Thr138 (HLA-C*04:01) and d2 as the distance between CA of Asp74 and Ala149 (HLA-C*04:01). Following 20 ns MD simulation on WFHDIPLPW-HLA-C*04:01, d1 increased from 9171 Å to 10,041 Å and d2 from 21,132 Å to 21,773 Å.

**Figure 7:**
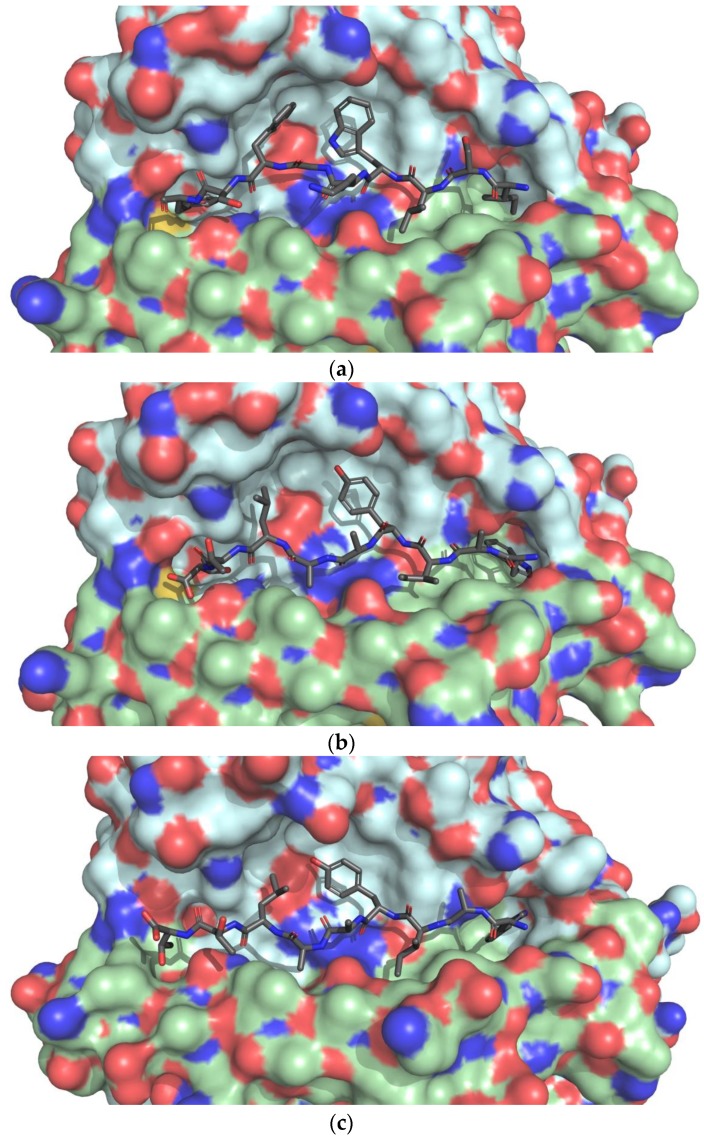
Conformational changes in MD simulation of WAIYAALTT- to HLA-DRA/DRB1*15:02. (**a**) Nine-mer core of a self-epitope from chain α3 type IV collagen (ISLWKGFSF), bound to HLA-DRA/DRB1*15:01 (PDB ID: 5V4M); (**b**) epitope WAIYAALTT docked to HLA-DRA/DRB1*15:02 (modeled from 5V4M); (**c**) epitope WAIYAALTT with HLA-DRA/DRB1*15:02 after 20 ns MD simulation. HLA chains α colored green and β blue.

In the case of WAIYAALTT, the MD simulation for 20 ns slightly shifted the position of Trp1 outward from the pocket with both hydrophobic rings still inside, and Ala2 was now closer to chain B, resulting in closer residue one to two conformations to the crystal structure (Figure 7a,c). Residues four and seven were also still located in the lateral pocket, and residues five and eight still pointed upward. The main difference was observed for Thr9, which dissociated itself from the HLA pocket and Thr8 that helped in accommodating the dissociation of Thr9.

### 3.6. Prediction of B-cell Linear Epitopes

An epitope search was performed for all Zika proteins: C, prM and M, E, Ns1, NS2A, NS2B, NS3, NS4A, 2K, NS4B, and NS5. Two programs were employed to search for B-cell linear epitopes. A list of consensus epitopes was first generated by combining the two prediction results. A total of 42 consensus epitopes were gathered. Among these, no epitopes were present for NS2A/B, NS4A, and 2K. The highest epitope densities were observed in NS1, NS3, and NS5. The list of consensus epitopes is available in Table S5 (Supplementary file).

Similar to T-cell epitopes, toxicity, conservancy and antigenicity tests, were performed on consensus epitopes, then their similarity to human proteins was checked. The results for these tests can be seen in Table S5. After the elimination process, 22 epitopes were found to be non-toxic, conserved, having antigenic residues and non-autoimmunity-inducing are displayed in Table 6. 

Lastly, each epitope was checked for the presence of a T-cell epitope residue, which will increase its quality as a vaccine epitope. The filtered epitopes were compared against epitopes for the 13 HLA alleles (both MHC-I and II) that had passed immunogenicity (MHC-I only), toxicity and conservancy tests. Epitopes EWFHDIP (E), WRDRYKYHPDSPR (NS1), and DVPAPKE (NS3) were found to share epitopic residues with MHC-I, whereas epitopes LDPYWGDVKQD and WMDARVCSDHA from NS3 shared epitopic residues with MHC-II.

### 3.7. Prediction of B-cell Conformational Epitopes

Initially, a PDB search for tertiary Zika protein structures was performed. The only usable tertiary structures came from proteins C, E, prM and M, NS1, NS2B, NS3 protease, NS3 helicase and NS5. No tertiary structure that could be used as a template for proteins NS2A, NS4A and NS4B was found. To use the American consensus sequence, proteins prM, NS3 (protease and helicase domains) and NS5 were modeled using homology modeling and NS1 using point mutation H1R. The list of proteins, reference structures and modeling methods are presented in Table 7. For structures generated using homology modeling, their validations can be seen in Table S6 (Supplementary file).

**Figure 8:**
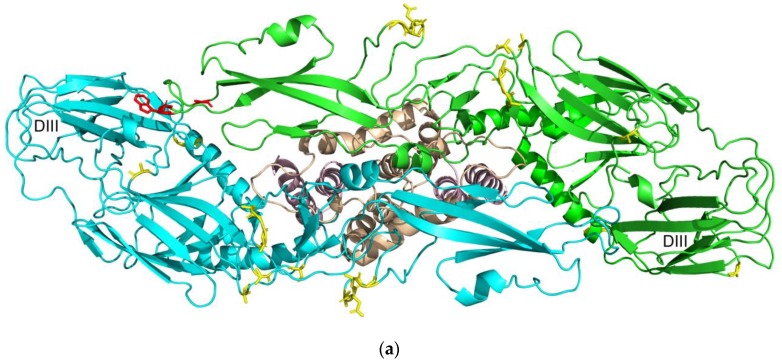

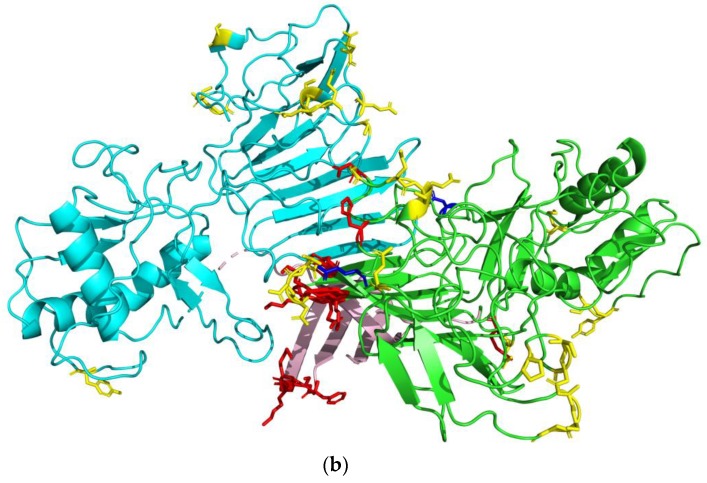
Conformational epitopes mapped to their respective proteins. Epitope residues are colored yellow. Epitopes only found in the monomeric structure are colored red, and epitopes only found in the multimeric structure are colored blue. (**a**) E protein (from above) in the form of E-M heterodimer. The chain used to find monomeric epitopes are colored green, transmembrane domains are colored pink and M proteins are colored tan; (**b**) NS1 protein as a dimer. The chain used to find monomeric epitopes are colored green, the beta-roll domain is colored pink.

Epitope prediction was first performed in its monomeric form, and then if possible as a biological assembly. The complete list of epitopes can be found in Table 8. The location of epitope residues for proteins E and NS1 is illustrated in Figure 8.

## 4. Discussion

### 4.1. Prediction of T-cell Epitopes Recognized by Class I and II MHC

After acquiring pathogenic gene mutations, the Zika virus reached America and caused an outbreak in the naïve population [5,6,7]. To start the screening of epitopes, an amino acid sequence is required (see Figure 1). In this study, sequences from the Zika virus currently circulating in America were used to find the sequence used in screening.

The specificity of each HLA’s epitope recognition [72] means that the selection of correct HLA molecules can affect the success of epitope screening. In this study, various HLAs common in Indonesia were used to screen for T-cell epitopes. Indonesia as a tropical country, has a very suitable climate for Zika mosquito vectors. Introduction of the more recent and dangerous American strains to this area can potentially trigger an outbreak. An example of this kind of introduction was in Singapore from a traveler returning from Brazil in 2016, although it did not trigger an outbreak [86].

Some HLAs have been associated with protection or vulnerability to certain diseases; examples include Dengue type 2 protective HLAs HLA-DRB1*04 and HLA-DRB1*07 that have been reported to have lower frequencies in the patient population than the healthy population [78]. Including protective HLAs in epitope screening are expected to return more essential epitopes for pathogen neutralization. Unfortunately, reports that associate certain HLAs with protection or vulnerability to Zika are not yet available. 

Screening for T-cell epitopes was done for all 26 MHC-I and II HLA molecules. A peptide was said to be epitope for its respective HLA if it was predicted to bind strongly to the HLA molecule. Two predictors were used in both finding class I MHC epitopes (HLA-A and B) and class II (HLA-DR and DQ) to generate consensus epitope-HLA pairs.

Consensus epitopes were then passed through several tests to ensure their quality as a vaccine component. For class I MHC, epitope immunogenicity was firstly evaluated. In this case, immunogenicity is defined as the ability of a peptide-MHC-I complex to bind with a T-cell receptor [40]. Peptide bound to MHC has specific residues facing outwards, giving a larger contribution to the interaction with TCR [45,87]. Toxicity was then assessed. Certain peptides can give a toxic reaction when administered by inhibiting certain biological functions [88]. After that, the epitope conservation analysis was done by considering the high specificity of MHC molecules and continuous mutation of viruses. It means a peptide used in a vaccine needs to be conserved among the various strains of the virus. An extended polyprotein sequence pool covering wider geographical area was generated to evaluate epitope conservancy. Epitopes were then put through the IEDB population coverage test [47] for regions vulnerable to Zika. This was done because HLA molecules are highly polymorphic with high peptide specificities [72], but their coding allele frequencies differ across various populations. To ensure the effectiveness of a vaccine in the general population, epitopes must be able to activate the desired immune response for the majority of the targeted population. 

When checking coverage for MHC-I, coverage numbers for Central America were very low (Table 4), and thus excluded when calculating averages. When checking for the MHC-II coverage, both chains were included and counted separately, resulting in bloated coverage numbers. 

Epitopes were then screened for the presence of antigenic residues. While this method is usually aimed towards B-cell epitope screening, T-cell epitopes may also come from digested B-cell epitopes [72]. Therefore, the T-cell epitope in an antigenic region is expected to have better potential of becoming a good epitope. Finally, high similarity between the epitope and parts of human proteins may raise concerns of an autoimmune response [89]. To minimize the risk of an autoimmune response, epitopes were subjected to BLASTp [49] against a human proteome. 

The remaining peptides have passed various tests to ensure their quality as a vaccine component. The peptides are predicted to be able to strongly interact with MHC molecules, and be physicochemically and biologically suitable for use in humans to safely elicit the desired immune response. The coverage analysis over various geographic regions also means that these peptides would be suitable for use in a larger population.

### 4.2. Molecular Docking of T-cell Epitopes to MHC Molecules

Mirza et al. [22] described the drawback in using rigid body docking, where both the receptor and ligand are not allowed to change their conformation. Since the peptides were modeled in unbound conformation, they tended to interact with themselves, forming folds or helices which shouldn’t be present in the bound conformation. These secondary structures were preserved in the docked structures from Mirza et al. [22]. However, since ligand flexibility was allowed in this study, a linear conformation could be found, solving the inherent problem associated with rigid body docking.

The structure of HLA used will affect the epitope docking result. To obtain the desired docking conformation, holo structures of receptors were used, with the assumption that in real conditions, both the HLA and epitope will adopt conformations similar to the holo structure. Most bound MHC ligands also have a similar conformation [72,90]. Therefore, bound ligands in receptor/reference structures were used to evaluate the correctness of docking results by visual inspection of backbone and side chains.

From nine docked conformations returned by AutoDock Vina [60], one conformation closest to the reference ligand was selected for analysis. Unfortunately, AutoDock Vina [60] returned highly varying conformations with many having a folded structure. This was more evident in MHC-II, where on average only one out of nine conformations could be considered similar to the reference (data not shown). The overall lack of docking quality may be caused by the program itself that is more specialized for docking of small ligands [60]. On the other hand, the high flexibility of peptides makes it a great challenge to produce a correct docking result [91,92].

In the case of MHC-II, receptors from homology modeling had an overall lower quality when compared to their reference structures (Table S2, Supplementary file), and since they were not modeled together with the ligand, some features of their binding sites could have been lost. The prediction accuracy for MHC-II epitopes is also still low [93], while the high prediction accuracy for MHC-I has been reported [94]. The poorer results for the MHC-II docking could also come from falsely predicted epitopes. Two particular cases are IEMAGPMAA and ILLVAHYM, where no conformation close to the reference ligand could be found. ILLVAHYM was even docked to a non-modeled receptor.

Inter-protein interactions are dominated with non-covalent interactions [95]. Hydrogen bond plays a significant role in stabilizing the ligand in its receptor, and its strength among non-covalent interactions makes it a main parameter in observing receptor-ligand interactions. Interestingly, the amounts of hydrogen bonds for these epitopes are still lower than many other epitopes.

More detailed analysis of each interaction is required to obtain a more accurate interpretation. The complete list of hydrogen bonds for all epitopes is available in Tables S3 and S4. Some atoms shared hydrogen bonds with several other atoms. Further inspection on WFHDIPLPW revealed two hydrogen bonds shared by one atom, while AIYAALTTF with 12 hydrogen bonds had seven hydrogen bonds shared by three atoms. The same phenomenon could not be observed for LYFHRRDLR (MHC-II, 12 hydrogen bonds) that only had two hydrogen bonds shared by one atom. Another important parameter to consider is the interaction distance that affects its strength. Directions of hydrogen bonds also make for interesting considerations since they influence the strength of the hydrogen bond [96]. Overall, these factors, including forces other than hydrogen bonds, are considered in the calculation of binding energy. The two epitopes with lowest binding energies WFHDIPLPW and WAIYAALTT were thus selected for further analysis by molecular dynamics simulation. 

### 4.3. Molecular Dynamics Simulations

In the context of epitope searching, molecular dynamics (MD) simulation can be used to observe the stability of a peptide-MHC complex and evaluate the quality of the epitope [85,97]. Overall, MHC molecules showed higher stability when bound to its ligand (epitope). Considering that the original crystal structures were of holo conformation, the higher degree of changes in unbound HLAs can be partially explained by the search of a new conformation, which would be their native apo structure. 

When correctly bound to HLA, a typical nonameric peptide would have the residue in each position interact with the HLA molecule in a manner specific to that HLA (an example is shown in Figure 6a) [72,90]. A known HLA-peptide complex structure can thus be used to evaluate the experimental epitope structure in the same/very similar HLA based on the position and orientation of each residue. The analysis of the crystal structure of HLA-Cw4 and its epitope (1QQD) showed that the peptide ligand had been known to be buried in positions one to three, seven and nine, with residues one, two, seven, and nine buried deep inside four pockets formed by the HLA molecule, whereas residues four and eight are the most exposed for interaction with receptor KIR2DL1 [84]. Residues three to four are also known to form a kink on the ligand structure [84], whereas the docked structure revealed residues two to three (FH) forming the kink (Figure 6b), which moved to residues seven to nine (LPW) following the MD simulation (Figure 6c). One possible reason being His3 that was facing outward in the docked structure. The opening of the receptor structure was also unexpected, as the closed ends of the class I HLA function to restrict most epitopes to eight to ten aa in length [72]. The opening of the -COOH end part of the receptor might be explained by the high density of residues seven to nine (LPW). The opening of the receptor structure was also shown by increasing d1 and d2 values, indicating a non-optimally bound epitope.

Looking at the characteristics of each residue, Trp1 is a large nonpolar residue, while HLA-Cw4 is known to accommodate smaller polar residues like Serine and Glutamine at position 1 [84]. Phenylalanine at position 2 is very similar to Tyrosine (Figure 7a), which both contain a hydrophobic ring. As expected, Phe2 was also buried deep in the same pocket as Tyr2 (Figure 7b,c). A small lateral pocket can accommodate smaller amino acids for residue three [38], but the side chain of His3 pointed upward, both before and after MD (Figure 7b,c). Residue seven is supposed to be buried in a pocket, but Leu7 pointed outward and Pro8 inward (Figure 7b,c), contrary to the crystal structure (Figure 7a) where residue seven is buried and eight exposed [84]. The pocket for residue nine is hydrophobic and was filled by Tryptophan. Following the MD simulation, the HLA residues Lys80 and Lys146 separated, and Tyr9, despite still facing inward was positioned higher (closer to the surface) (Figure 7c). Overall, it appeared that the ligand tended to separate itself from the HLA molecule, most visibly in residues six to eight, leaving Phe2 as the main anchor residue after 20 ns of MD simulation. However, it is not yet known whether this phenomenon was caused by incorrect epitope residues or improper docking result.

As for WAIYAALTT, the receptor structure HLA-DRA/DRB1*15:02 was obtained by the V115G point mutation of chain B of HLA-DRA/DRB1*15:01 (PDB ID: 5V4M) [98]. The conformational analysis was done using the template structure. Amino acids of residues one, four, seven, and nine were reported to be buried in HLA pockets, working as main anchors [98]. In the docked structure, Trp1 and Trp9 were buried, and Tyr4 and Leu7 pointed toward the lateral pocket as described [98] (Figure 8b). The similar orientations of ligand residues indicate a good docking result. 

The discharge of residue nine from its pocket possible indicates an incompatibility with interacting HLA residues. Threonine itself is a polar amino acid, which goes against the accepted residues in the ninth position, Phe, Leu, Val, and Ile, all nonpolar [98]. During the simulation, Thr9 might have gone up to interact with water molecules. The dramatically change in the Thr9 position also explains the high RMSF values in residues eight and nine (Figure 5b). 

All things considered, the docked epitope WAIYAALTT is sufficiently good for residues one to eight when docked to HLA*C:04:02. However, residue nine should not have been polar. Whether threonine in the ninth position makes this epitope, a bad epitope is yet to be determined. In this respect, however, the simulated structures from the result of each 20 ns of MD simulation analyses cannot be conclusive but open the way to further research developments.

### 4.4. Prediction of B-cell Linear Epitopes

Several programs have been developed to predict B-cell epitopes from a protein sequence. Unfortunately, the prediction capabilities of these programs are still rather poor [50]. The linear B-cell epitope was searched for on all Zika proteins. Despite not all proteins being readily exposed for binding with the antibody, an epitope search for non-naturally-exposed proteins would still be useful for physiological laboratory testing, as was done by Saisawang et al. [99].

Interestingly, the removal of pr sequence altered BepiPred-2.0 scoring for the M protein, giving rise to a new epitope previously undetected in prM. Care should be taken regarding the completeness of the sequence given for BepiPred-2.0.

The elimination method used in this study had several shortcomings where longer epitopes tended to be eliminated in the conservancy test, and shorter epitopes tended to be eliminated in the autoimmunity test. Even so, mitigating these problems would require a variation of epitope lengths, such as trimming into several parts or inclusion of neighboring residues. While in vaccine/antibody design modifications may be required, each epitope was treated as one peptide for simplicity reasons.

Despite no epitope having both MHC-I and II epitope residues, five epitopes that share epitopic residues with MHC have better potential as a good linear epitope due to cross-recognition by T-cells. Of particular interest are those recognized by CD4+ T-cells that help in B-cell activation [72]. Among these epitopes, care should be taken before employing DVPAPKE for use in humans, as BLASTp found only one residue difference; lysine from its human counterpart arginine, which are both similar (hydrophilic and positively charged). A conservative mutation, as in this case, would result in minimal characteristic change [100]. Peptide extension might solve this issue. 

In conclusion, all 22 filtered consensus epitopes have the potential to become a linear epitope for B-cells. Among them, five epitopes, EWFHDIP (E), WRDRYKYHPDSPR (NS1), DVPAPKE (NS3), LDPYWGDVKQD (NS3) and WMDARVCSDHA (NS3) are the most potential candidates owing to the presence of T-cell epitope residues. Care should, however, be taken for DVPAPKE for its high similarity to the human protein.

### 4.5. Prediction of B-cell Conformational Epitopes

Despite conformational epitopes making up 90% of B-cell epitopes [101], predicting these epitopes is much more difficult due to the need of the protein tertiary structure. To create a vaccine for humans, the main target proteins are those naturally exposed for interaction with B-cell antibodies. In addition, the organization of proteins can change depending on the condition. In virions, the E protein is an exposed protein that plays a role in cellular entry [102]. Before maturation, prM-E complexes form trimeric spikes [102,103]. In mature virions, following a successful prM cleavage, these M-E complexes take on a dimeric arrangement on the surface of the virus, embedded at their transmembrane domains (shown in Figure 8a) [104]. Another protein, NS1, is the only nonstructural protein secreted out of the cell [105], allowing antibody attachment. Inside cells, NS1 forms dimers but secreted NS1 forms hexamers in the configuration of three dimers [105]. In both forms, the beta-roll domain (shown in Figure 8b) is not exposed because it associates with the membrane or is hidden inside, forming a hydrophobic core [105]. This information means antibodies are unlikely to bind to the ‘underside’ of proteins E (which contains the transmembrane domain) and NS1 (which contains the beta-roll domain), particularly in humans. Aside from proteins E and NS1, C also forms a dimer, and its presence as a monomeric and trimeric protein has been reported [106]. Even though in laboratories, the research environment can be manipulated, in human applications, information regarding how the proteins associate becomes essential. Therefore, in epitope prediction, more focus was given to proteins E and NS1 that can be naturally bound by antibodies.

The search for E protein epitopes was done for both its monomeric and E-M heterodimer forms and reported in Figure 8a. Chain E was used to represent monomeric epitopes since it returned to more epitope positions. Glycosylation in Asn154 has been reported to play a role in vivo Zika infectivity by inhibiting the production of reactive oxygen, which plays a role in mosquito immunity [106]. Unfortunately, glycosylation data did not affect scoring. Thus, its effect could not be observed. In human applications, E proteins exposed for antibody binding will mostly be found on the virion surface in the form of an E-M heterodimer [104]. Hence these heterodimer epitopes can be focused on vaccine/antibody design. It should also be noted that E proteins are packed with the side containing the transmembrane domain (Figure 8a, pink) facing inward, hidden from the reach of antibodies [104].

Most known Zika antibody-antigen complex structures come from the E protein [107], so it is interesting to compare the epitopes predicted by DiscoTope with the empirical data available. Compared with available antibody-antigen structures, the E fusion loop region, which was predicted as the epitope only in the monomeric structure, is also a target for a flavivirus broadly neutralizing antibody 2A10G6 [108]. Some antibodies like Z20 and ZIKV-117 have been reported to work on the dimeric form of the E protein, attaching themselves across both monomers [109,110]. Predicted epitope residues Asp320-Gly232 are also part of the ZIKV-117 binding site [109], and residue Gly232 is a part of the Z20 binding site [110]. However, these antibodies still have extensive interactions with many surrounding residues. Lastly, domain III (labeled in Figure 8a) is also known to be a binding site for several antibodies [111,112]. Only one residue (Gln350) was predicted as the epitope in this domain, which is known to interact with antibody Z006 [111]. As is the case with Z20 and ZIKV-117, this residue is only a part of a much larger interaction site. Due to the large surface area of antibodies, antigens can be expected to interact with antibodies in several residues. In the latter three cases, the predicted epitopic residues are not located at the ‘center’ of the actual epitopes. 

The NS1 protein was also checked in both its monomeric and dimeric form (Table 8, Figure 8b). As with the E protein, this protein can also be found outside of cells, in the form of hexamers, which model can be found in [104]. For the creation of the vaccine/antibody, the side containing beta-roll domain (Figure 8b, pink) will be hidden and unlikely for antibody binding [105]. Interestingly, two epitopes Asp157 and Glu258 were present in the dimeric but not monomeric structure. Asp157 is positioned close to the β-roll and β-ladder of the other chain, and the appearance of Glu258 may be caused by the wing of the other chain. In both cases, these epitopes appeared because of the presence of new neighboring residues previously absent in the monomeric structure. 

Aside from the two proteins accessible extracellularly under natural conditions, the epitope search for other proteins, such as nonstructural proteins, is also interesting for its usefulness in laboratory testing. The epitope search for the C protein revealed several epitopes found only in its monomeric form, whereas no epitope was detected for its multimeric form (Table 8. The prM protein spans across the entire E protein and the consecutive epitopic residues form a long stretch (Table 8). This stretch might not be a true epitope since the E protein was not accounted for during prediction. The M protein in the form of the E-M heterodimer did not have any epitope, but by itself, a long stretch of epitopic residues was found (Table 8). This epitope is located at the end of the long stretch of the prM epitope. The structure for NS2B was obtained from a complexed structure with the protease domain of dengue NS3 [113]. NS2B is a cofactor of the NS3 protease and circles the protein [113], similar to the case of prM; the long ‘stretch’ was also identified as the epitope (Table 8). In the presence of NS3 it is also probable that this epitope will disappear. Epitope residues for the NS3 protease and helicase are available in Table 8. Unfortunately, the complete structure of NS3 has not been determined, forbidding the epitope search on the full protein structure. Epitopes for the last protein NS5 are displayed in Table 8. This protein gave the most epitopic residues, partly from its large size.

Epitope prediction using DiscoTope 2.0 [114] uses spatial information to predict epitopic residues. The predicted epitopes tend to be in the more exposed areas. The structural unit used will also influence prediction results since some residues will become available/unavailable for interaction with antibodies. For the application in humans, it is important to select the correct structural unit in predicting conformational epitopes. The epitopic regions potential as antibody targets, or can be said to activate B-cells for proteins E and NS1 in their extracellular forms have been predicted. Some predicted epitopic residues in the E protein have also been confirmed to interact with antibodies [108,109,110,111]. Additionally, B-cell epitope residues have been predicted for proteins C, prM, M, NS2B, NS3 protease and helicase, and NS5. A similar study of immunoinformatics approach in the development of the novel peptide vaccine using T- and B-cells highly conserved epitopes of Zika envelope glycoprotein showed positive results as a potential vaccine [53].

Further investigation on how the immunogenic peptide could induce T-cell responses by Paquin–Proulx et al. (2017). An interesting result showed that T-cell cross-reactivity could give a positive impact on the ZIKV infection in vitro. Paquin–Proulx et al. successfully demonstrated that the ZIKV CD4+ T-cell response induced by DENV vaccination could promote the appearance of cross-reactive antibodies mediating ADE. However, a better understanding of the interactions between the ZIKV and DENV immunity, such as induction by vaccination or by natural infection, will be necessary to provide safe and efficient vaccines [115]. Several studies have reported how the computational prediction of antigenic peptides were successfully validated experimentally to combat HIV-1, malaria parasites and leishmaniasis, a vector-borne parasitic disease [116,117,118]. In validating the predicted epitopes, it is suggested to integrate genomics with proteomics approaches to represent a valid strategy in refining the antigen characterizations as well as give the useful insights on abundance and subcellular localization of pathogenic antigens [119]. These predicted epitopes need further investigation by synthesizing and evaluating in vitro using the cell culture or rodent model to determine the impacts of immunity to ZIKV.

## 5. Conclusions

By only using necessary peptides, the peptide vaccine is expected to minimize risks associated with unnecessary antigens. Furthermore, using in silico methods to screen for the best candidates can significantly reduce the time and materials spent in laboratories. In this study, the in silico search has been performed to find peptides that can potentially trigger the immune system, and be a component of the Zika vaccine. For the T-cell, the search method was adjusted for certain regions and their surrounding populations. Potential epitopes for both MHC-I and MHC-II have been screened for from the complete Zika protein sequence. Docking and molecular dynamics simulations have also been performed on the most promising epitopes. Regrettably, MD results were unsatisfactory for the best MHC-I epitope, WFHDIPLPW, which was probably caused by an improper starting docked structure. MD results for the best MHC-II epitope WAIYAALTT were quite promising apart from the harmful effect of one residue’s polarity.

Coverage analysis does not account for HLA allele pairing, whereas for some HLA both chains are required to confer their specificity. Finding the frequencies of the allele pairs (chain A and B) is the ideal solution, but it is difficult to do. In this case, the epitope search using globally common alleles might give a better result. 

The linear and conformational epitopes of the B-cell have been successfully mapped. Several promising B-cell epitopes that have been verified by physicochemical modeling have been reported in this study. Most promising among them are EWFHDIP from the E protein, WRDRYKYHPDSPR from the NS1 protein, and DVPAPKE, LDPYWGDVKQD and WMDARVCSDHA from the NS3 protein, which are also predicted as T-cell epitopes. Of particular interest are LDPYWGDVKQD and WMDARVCSDHA since they would also be recognized by T helper cells which play a role in B-cell activation. Caution should be taken before using DVPAPKE for its similarity to a part of the human proteome.

Conformational epitopes for the B-cell have also been searched for, for proteins C, E, prM, M, NS1, NS2B, NS3 (protease and helicase domains) and NS5. Particular attention was given to the proteins E and NS1 that can be found as an extracellular matrix under native conditions. These proteins are the primary antibody targets in the human body. This study also considered the change in epitope residues when a protein is in its monomeric or multimeric form.

## Figures and Tables

**Table 1:** Human leukocyte antigen (HLA) alleles for T-cell epitope mapping the common and protective to dengue virus (DENV).

HLA Class	HLA Allele	Common	Protective	References
I	HLA-A*02:01	✓		[73]
	HLA-A*03:01		✓	[74]
	HLA-A*11:01	✓		[73]
	HLA-A*24:02	✓		[73]
	HLA-A*24:07	✓		[73]
	HLA-A*33:01		✓	[75]
	HLA-A*33:03	✓		[73]
	HLA-B*15:02	✓		[73]
	HLA-B*15:13	✓		[73]
	HLA-B*18:01		✓	[74]
	HLA-B*35:01		✓	[74,76]
	HLA-B*35:05	✓		[73]
	HLA-B*44:03	✓		[73]
II	HLA-DRB1*03:01	✓		[73]
	HLA-DRB1*04:01		✓	[77,78]
	HLA-DRB1*07:01	✓	✓	[73,78,79]
	HLA-DRB1*09:01		✓	[80]
	HLA-DRB1*12:02	✓		[73,79]
	HLA-DRB1*15:01	✓		[73]
	HLA-DRB1*15:02	✓		[73,79,81]
	HLA-DQA1*01:01	✓		[79]
	HLA-DQA1*01:02	✓		[79]
	HLA-DQA1*06:01	✓		[79]
	HLA-DQB1*03:01	✓		[79,81]
	HLA-DQB1*05:01	✓		[79,81]

**Table 2:** HLA alleles for T-cell epitope mapping.

Pos	Protein	Epitope	NA	SA	SAS	SEA	Oce	WI	WAf	CAf	CA	Avg ^1^	Auto-Immunity ^2^
2310	NS4B	AIYAALTTF	81.62	63.67	75.25	81.47	78.14	66.07	75.72	85.91	2.97	75.98	○
1488	NS2B	FAAGAWYVY	81.84	73.23	76.47	74.99	56.09	75.05	78.11	80.77	7.02	74.57	○
2862	NS5	IAMTDTTPY	72.99	50.65	69.71	75.12	62.86	71.10	64.16	74.85	6.44	67.68	○
664	Env	MMLELDPPF	92.11	89.51	77.07	91.02	83.26	85.29	84.11	89.86	5.70	86.53	○
46	Capsid	MVLAILAFL	89.18	81.56	74.70	63.25	59.63	81.05	87.53	93.37	7.95	78.78	○
1744	NS3	RYMTTAVNV	80.88	70.87	76.55	78.60	81.71	41.77	79.54	81.32	2.19	73.91	○
507	Env	WFHDIPLPW	74.92	68.36	60.35	80.50	79.53	53.02	69.01	70.19	3.75	69.49	○
2996	NS5	WYMWLGARF	74.49	67.56	57.78	77.88	75.46	59.09	70.34	70.83	1.39	69.18	○
2356	NS4B	YAWDFGVPL	97.13	92.59	90.73	92.52	84.57	81.90	94.71	94.86	2.98	91.13	○
2997	NS5	YMWLGARFL	89.75	86.09	77.98	78.90	69.37	54.85	90.41	84.46	0.00	78.98	○

^1^ Average numbers for major histocompatibility complex-I (MHC-I) epitopes do not include Central America. ^2^ O—Pass. X—Does not pass; may cause autoimmunity. Note: Region abbreviations: NA—North America, CA—Central America, SA—South America, SAS—South Asia, SEA—Southeast Asia, Oce—Oceania, WI—West Indies, WAf—West Africa, CAf—Central Africa. Population coverage numbers are in percentage.

**Table 3:** Shortlisted ten major histocompatibility complex-II (MHC-II) epitopes, with coverage values and autoimmune induction potential.

Pos	Protein	Epitope	NA	CA	SA	SAS	SEA	Oce	WI	WAf	CAf	Avg	SB	WB	SB + (WB/2)	Auto-Immunity ^1^
84	Capsid	FKKDLAAML	100.00	99.98	99.78	99.82	89.63	98.77	60.73	99.73	99.83	94.25	10	9	14.5	○
2445	NS4B	IAVAVSSAI	99.46	100.00	99.79	99.29	96.87	99.21	99.13	99.82	97.47	99.00	11	9	15.5	○
1398	NS2B	IEMAGPMAA	100.00	100.00	100.00	99.72	99.44	99.93	99.70	99.94	99.71	99.83	7	7	10.5	○
2382	NS4B	IILLVAHYM	99.95	99.19	98.50	97.04	74.51	94.90	71.01	87.61	92.44	90.57	5	11	10.5	○
2396	NS4B	LQAAAARAA	100.00	99.99	99.91	99.35	98.89	99.91	97.86	99.42	99.29	99.40	11	2	12	×
3287	NS5	LYFHRRDLR	99.94	99.05	96.53	96.46	62.07	90.11	69.54	89.41	93.30	88.49	9	3	10.5	○
2335	NS4B	SLMAMATQA	99.00	99.93	99.64	98.43	98.56	99.37	99.94	99.95	99.13	99.33	11	7	14.5	○
557	Env	TALAGALEA	99.74	100.00	100.00	98.00	99.19	99.80	99.32	99.51	98.91	99.39	10	2	11	×
2309	NS4B	WAIYAALTT	99.81	100.00	99.89	98.86	99.79	99.83	98.60	99.61	98.61	99.44	6	11	11.5	○
427	Env	YRIMLSVHG	99.95	92.92	96.84	97.70	86.77	97.30	88.96	98.29	95.73	94.94	11	5	13.5	○

^1^ O—Pass, X—Does not pass; may cause autoimmunity. Note: Region abbreviations: NA—North America, CA—Central America, SA—South America, SAS—South Asia, SEA—Southeast Asia, Oce—Oceania, WI—West Indies, WAf—West Africa, CAf—Central Africa. Population coverage numbers are in percentage.

**Table 4:** Summary of docking results of epitope with MHC-I.

Protein	Peptide	HLA	Receptor	Binding Energy (kcal/mol)	Ki	#H Bond
Capsid	MVLAILAFL	A*02:01	2GIT	−9	4.52 × 10^−7^	9
NS5	YMWLGARFL	A*02:01	2GIT	−9.3	2.78 × 10^−7^	8
NS4B	YAWDFGVPL	A*02:01	2GIT	−10.3	5.48 × 10^−8^	7
NS3	RYMTTAVNV	A*24:02	2X4O	−8.9	5.32 × 10^−7^	9
NS5	WYMWLGARF	A*24:02	2X4O	−7.5	5.16 × 10^−6^	7
NS4B	AIYAALTTF	B*15:01	5TXS	−9.9	1.05 × 10^−7^	12
NS2B	FAAGAWYVY	B*35:01	3LKN	−10.1	7.58 × 10^−8^	9
NS5	IAMTDTTPY	B*35:01	3LKN	−9.8	1.23 × 10^−7^	8
Env	WFHDIPLPW	C*04:01	1QQD	−11	1.76 × 10^−8^	8

**Table 5:** Summary of docking results of epitope with MHC-II.

Protein	Peptide	HLA	Receptor ^1^	Binding Energy (kcal/mol)	Ki	#H Bond
NS4B	IAVAVSSAI	DQA1*05:01/DQB1*03:03	1UVQ*	−6.8	1.61 × 10^−5^	4
NS2B	IEMAGPMAA	DQA1*05:01/DQB1*03:03	1UVQ*	−6.3	3.62 × 10^−5^	4
NS4B	SLMAMATQA	DQA1*02:01/DQB1*03:03	1UVQ*	−7.3	7.14 × 10^−6^	6
Env	YRIMLSVHG	DRB1*11:01	4MD5*	−7.3	7.14 × 10^−6^	9
NS5	LYFHRRDLR	DRB1*03:01	1A6A	−8.5	1.02 × 10^−6^	12
NS4B	IILLVAHYM	DRB1*15:01	1BX2	−6.5	2.62 × 10^−5^	6
Capsid	FKKDLAAML	DRB1*09:01	5V4M*	−8	2.29 × 10^−6^	5
NS4B	WAIYAALTT	DRB1*15:02	5V4M*	−10.4	4.66 × 10^−8^	7

^1^ The receptor with an asterisk sign (*) indicates that the structure was used as a template in modeling.

**Table 6:** Linear epitopes that passed toxicity, conservancy, antigenicity and autoimmunity tests.

Protein	Position	Length	Peptide ^1^
prM	56	10	LDEGVEPDDV
	84	20	KKGEARRSRRAVTLPSHSTR
M	25	7	YTKHLIR
E	89	15	QYVCKRTLVDRGWGN
	146	8	SQHSGMIV
	216	7	EWFHDIP
	428	9	AWDFGSVGG
NS1	28	13	WRDRYKYHPDSPR
	141	8	KECPLKHR
	338	11	RKEPESNLVRS
NS3	6	7	DVPAPKE
	27	7	TRRLLGS
	65	11	LDPYWGDVKQD
	82	9	PWKLDAAWD
	594	11	WMDARVCSDHA
NS5	34	15	EVCREEARRALKDGV
	153	9	SPEVEEART
	247	17	PRRPVKYEEDVNLGSGT
	353	9	QRVFKEKVD
	363	7	RVPDPQE
	414	24	FEEEKEWKTAVEAVNDPRFWALVD
	598	9	QDQRGSGQV

^1^ Underlined residues are predicted as antigenic. Epitope residues for MHC-I are colored in red, and for MHC-II in blue.

**Table 7:** Accession, origin and isolation year for Zika polyprotein sequences used to obtain consensus sequence.

Protein	PDB ID
C	5YGH
E	5IRE
prM	4B03 via homology modeling
M	5IRE
NS1	5GS6 via point mutation: H1R
NS2B	5YOF
NS3 (protease domain)	5YOF via homology modeling
NS3 (helicase domain)	5VI7 via homology modeling
NS5	5U0B via homology modeling

**Table 8:** Predicted Zika conformational epitopes.

Protein	Epitope Residues ^1^
C	Chain A*: PRO26, PHE27, LYS74, LYS75, ASN96, ALA97, ARG98
E	Chain C: ASN52, SER129, GLU133, THR156, ALA229, ASP230, THR231, GLY232, ASP278, GLY279, ALA280, THR405, ILE407 Chain E*: ASN52, TRP101, GLY102, GLY109, GLU133, THR156, ALA229, ASP230, THR231, GLY232, ASP278, GLY279, ALA280, GLN350, THR406, ILE407
NS1	Chain A*: PHE8, SER9, LYS10, LYS11, ASP30, ARG31, LYS116, SER121, TYR122, ASP157, GLY190, LYS191, GLU192, GLU205, LYS206, ASN207, ASP208, THR209, TRP210, ASP234, GLY235, GLU237, GLU238, SER239, HIS253, GLU258, ALA303, SER304, GLY305, GLU315, PRO341, SER343, ASN344 Chain B: TYR122, GLY235, GLU237, GLU238, SER239, GLU258, ALA303, SER304, GLY305, GLU315, SER343
prM	HIS82, HIS83, LYS84, LYS85, GLY86, GLU87, ALA88, ARG89, ARG90, SER91, ARG92, ARG93, ALA94, VAL95, THR96, LEU97, PRO98, SER99, HIS100, SER101, THR102, ARG103, LYS104, LEU105, GLN106, THR107, ARG108, SER109, GLN110, THR111, TRP112, LEU113, GLU114, SER115, ARG116, GLU117, TYR118, THR119, LYS120, HIS121
M	ALA1, THR 3, LEU4, PRO5, SER6, HIS7, SER8, THR9, ARG10, LYS11, LEU12, GLN13, THR14, ARG15, SER16, GLN17, THR18, TRP19, LEU20, GLU21, SER22, ARG23, GLU24, TYR25, THR26, LYS27
NS2B	ASP50, MET51, TYR52, ILE53, GLU54, ARG55, ALA56, GLY57, ASP58, ILE59, THR60, TRP61, GLU62, LYS63, ASP64, ALA65, GLU66, VAL67, THR68, GLY69, ASN70, SER71, PRO72, ARG73, GLU80, GLY82
NS3-P	ARG13, ARG14, LEU15, LEU16, GLY17, GLY46, GLU47, ASP51, LYS104, ASP105, GLU156
NS3-H	ARG147, ASP148, ASP152, SER153, ASN154, SER155, PRO156, ILE157, MET158, ASP159, THR160, SER180, GLY181, LYS182, LYS223, HIS224, GLN225, GLU226, LYS243, ASP245, GLY285, ARG286, ASN287, PRO288, ASN289, LYS290, PRO291, GLY292, ASP293, TRP405, HIS408, GLY409, GLU410, LYS411
NS5	ALA10, ASN13, GLN14, MET15, SER16, ALA17, LEU18, GLU19, PHE20, TYR21, SER22, TYR23, LYS24, LYS25, SER26, GLY27, GLY103, PRO104, GLY105, GLN234, LEU237, GLY238, MET240, ASP241, GLY242, PRO243, ARG244, ARG245, PRO246, VAL247, LYS248, TYR249, SER264, CYS265, ALA266, GLU267, ALA268, ASN270, MET271, LYS272, GLU279, ARG282, ALA286, GLU287, THR288, TRP289, PHE290, PHE291, GLU293, TYR297, ARG298, THR299, TRP300, ALA301, TYR302, GLY304, TYR306, GLU307, ALA308, PRO309, THR310, SER313, ALA314, ASP342, THR343, THR344, GLN348, VAL351, PHE352, LYS353, GLU354, LYS355, VAL356, ASP357, THR358, ARG359, VAL360, PRO361, ASP362, LYS384, HIS385, MET452, GLY453, GLN459, LYS466, GLY467, SER468, ARG469, ARG521, ILE522, PRO523, GLY525, ARG526, ALA533, GLY534, LEU578, LYS583, GLY584, LYS585, THR586, GLU625, GLU628, MET629, GLN630, TRP633, LEU634, ARG636, ARG637, GLU639, ASN643, GLN646, SER647, ASP669, ASP670, ARG671, HIS674, LYS687, ASP688, THR689, GLN690, GLU691, TRP692, LYS693, PRO694, THR696, ASP699, ASN700, HIS715, LEU716, LYS717, ASP718, GLY719, ARG720, SER721, ILE816, GLU817, GLU818, ASP820, MET822, GLU823, ASP824, LYS825, THR826, PRO827, THR829, GLY838, LYS839, ARG840, GLY850, ARG852

^1^ For proteins checked in their multimeric form, the chain used in finding monomeric epitopes are marked by *. Epitopes found only in the monomeric structure are colored red, while epitopes found only in the multimeric structure are colored blue.

## Supplementary Materials

Supplementary materials can be found at https://www.mdpi.com/2076-2607/7/8/226/s1. Table S1: Zika polyprotein sequences used to obtain consensus sequence and in epitope conservancy analysis, Table S2: Validation of homology modeled MHC structures and their template structures, Table S3: List of hydrogen bonds for docked MHC-I epitopes, Table S4: List of hydrogen bonds for docked MHC-II epitopes, Table S5: List of consensus linear B cell epitopes, scores averaged across all residues, and their filtration results, Table S6: Validation of homology modeled MHC structures and their template structures, Diagram S1: Consensus Zika polyprotein sequence.

## References

[1] Gong Z., Gao Y., Han G.-Z. (2016). Zika Virus: Two or Three Lineages?. Trends Microbiol..

[2] Shen S., Shi J., Wang J., Tang S., Wang H., Hu Z., Deng F. (2016). Phylogenetic analysis revealed the central roles of two African countries in the evolution and worldwide spread of Zika virus. Virol. Sin..

[3] Plourde A.R., Bloch E.M. (2016). A Literature Review of Zika Virus. Emerg. Infect. Dis..

[4] Slenczka W. (2016). Zika Virus Disease. Microbiol. Spectr..

[5] Schuler-Faccini L. (2016). Possible Association Between Zika Virus Infection and Microcephaly—Brazil, 2015. MMWR Morb. Mortal. Wkly. Rep..

[6] World Health Organization Zika Situation Report 5 February 2016: Neurological Syndrome and Congenital Anomalies. http://www.who.int/emergencies/zika-virus/situation-report/5-february-2016/en/.

[7] Yuan L., Huang X.-Y., Liu Z.-Y., Zhang F., Zhu X.-L., Yu J.-Y., Ji X., Xu Y.-P., Li G., Li C. (2017). A single mutation in the prM protein of Zika virus contributes to fetal microcephaly. Science.

[8] Metsky H.C., Matranga C.B., Wohl S., Schaffner S.F., Freije C.A., Winnicki S.M., West K., Qu J., Baniecki M.L., Gladden-Young A. (2017). Zika virus evolution and spread in the Americas. Nature.

[9] Zanluca C., Melo V.C.A.D., Mosimann A.L.P., Santos G.I.V.D., Santos C.N.D.D., Luz K. (2015). First report of autochthonous transmission of Zika virus in Brazil. Mem. Inst. Oswaldo Cruz.

[10] De Oliveira W.K., de França G.V.A., Carmo E.H., Duncan B.B., de Souza Kuchenbecker R., Schmidt M.I. (2017). Infection-related microcephaly after the 2015 and 2016 Zika virus outbreaks in Brazil: A surveillance-based analysis. Lancet.

[11] (2017). Secretaria de Vigilância em Saúde—Ministério da Saúde Monitoramento dos casos de dengue, febre de chikungunya e febre pelo vírus Zika até a Semana Epidemiológica 25, 2017. Bol. Epidemiol..

[12] Lagunas-Rangel F.A., Viveros-Sandoval M.E., Reyes-Sandoval A. (2017). Current trends in Zika vaccine development. J. Virus Erad..

[13] Ortiz J.F., MacDonald M.L., Masterson P., Uversky V.N., Siltberg-Liberles J. (2013). Rapid evolutionary dynamics of structural disorder as a potential driving force for biological divergence in flaviviruses. Genome Biol. Evol..

[14] Tirado S.M.C., Yoon K.-J. (2003). Antibody-dependent enhancement of virus infection and disease. Viral Immunol..

[15] Guzman M.G., Vazquez S. (2010). The Complexity of Antibody-Dependent Enhancement of Dengue Virus Infection. Viruses.

[16] Cunha-Neto E., Rosa D.S., Harris P.E., Olson T., Morrow A., Ciotlos S., Herst C.V., Rubsamen R.M. (2017). An Approach for a Synthetic CTL Vaccine Design against Zika Flavivirus Using Class I and Class II Epitopes Identified by Computer Modeling. Front. Immunol..

[17] Zellweger R.M., Eddy W.E., Tang W.W., Miller R., Shresta S. (2014). CD8+ T-cells prevent antigen-induced antibody-dependent enhancement of dengue disease in mice. J. Immunol..

[18] Paquin-Proulx D., Leal F.E., Silveira C.G.T., Maestri A., Brockmeyer C., Kitchen S.M., Cabido V.D., Kallas E.G., Nixon D.F. (2017). T-cell responses in individuals infected with Zika virus and in those vaccinated against Dengue virus. Pathog. Immun..

[19] Alam A., Ali S., Ahamad S., Malik M.Z., Ishrat R. (2016). From ZikV genome to vaccine: In silico approach for the epitope-based peptide vaccine against Zika virus envelope glycoprotein. Immunology.

[20] Dar H., Zaheer T., Rehman M.T., Ali A., Javed A., Khan G.A., Babar M.M., Waheed Y. (2016). Prediction of promiscuous T-cell epitopes in the Zika virus polyprotein: An in silico approach. Asian Pac. J. Trop. Med..

[21] Dikhit M.R., Ansari M.Y., Vijaymahantesh, Kalyani, Mansuri R., Sahoo B.R., Dehury B., Amit A., Topno R.K., Sahoo G.C. (2016). Computational prediction and analysis of potential antigenic CTL epitopes in Zika virus: A first step towards vaccine development. Infect. Genet. Evol..

[22] Mirza M.U., Rafique S., Ali A., Munir M., Ikram N., Manan A., Salo-Ahen O.M.H., Idrees M. (2016). Towards peptide vaccines against Zika virus: Immunoinformatics combined with molecular dynamics simulations to predict antigenic epitopes of Zika viral proteins. Sci. Rep..

[23] Pradhan D., Yadav M., Verma R., Khan N.S., Jena L., Jain A.K. (2017). Discovery of T-cell Driven Subunit Vaccines from Zika Virus Genome: An Immunoinformatics Approach. Interdiscip. Sci. Comput. Life Sci..

[24] Yadav G., Rao R., Raj U., Varadwaj P. (2017). Computational modeling and analysis of prominent T-cell epitopes for assisting in designing vaccine of ZIKA virus. J. Appl. Pharm. Sci..

[25] Soria-Guerra R.E., Nieto-Gomez R., Govea-Alonso D.O., Rosales-Mendoza S. (2015). An overview of bioinformatics tools for epitope prediction: Implications on vaccine development. J. Biomed. Inform..

[26] Systèmes D. (2016). BIOVIA Discovery Studio Modeling Environment.

[27] Schrödinger L.L.C. (2018). The PyMOL Molecular Graphics System.

[28] Stranzl T., Larsen M.V., Lundegaard C., Nielsen M. (2010). NetCTLpan: Pan-specific MHC class I pathway epitope predictions. Immunogenetics.

[29] Hoof I., Peters B., Sidney J., Pedersen L.E., Sette A., Lund O., Buus S., Nielsen M. (2009). NetMHCpan, a method for MHC class I binding prediction beyond humans. Immunogenetics.

[30] Nielsen M., Lundegaard C., Lund O., Keşmir C. (2005). The role of the proteasome in generating cytotoxic T-cell epitopes: Insights obtained from improved predictions of proteasomal cleavage. Immunogenetics.

[31] Peters B., Bulik S., Tampe R., Van Endert P.M., Holzhütter H.-G. (2003). Identifying MHC class I epitopes by predicting the TAP transport efficiency of epitope precursors. J. Immunol..

[32] Moutaftsi M., Peters B., Pasquetto V., Tscharke D.C., Sidney J., Bui H.-H., Grey H., Sette A. (2006). A consensus epitope prediction approach identifies the breadth of murine T(CD8+)-cell responses to vaccinia virus. Nat. Biotechnol..

[33] Nielsen M., Andreatta M. (2016). NetMHCpan-3.0; improved prediction of binding to MHC class I molecules integrating information from multiple receptor and peptide length datasets. Genome Med..

[34] Andreatta M., Nielsen M. (2016). Gapped sequence alignment using artificial neural networks: Application to the MHC class I system. Bioinform. Oxf. Engl..

[35] Nielsen M., Lundegaard C., Worning P., Lauemøller S.L., Lamberth K., Buus S., Brunak S., Lund O. (2003). Reliable prediction of T-cell epitopes using neural networks with novel sequence representations. Protein Sci. Publ. Protein Soc..

[36] Peters B., Sette A. (2005). Generating quantitative models describing the sequence specificity of biological processes with the stabilized matrix method. BMC Bioinform..

[37] Sidney J., Assarsson E., Moore C., Ngo S., Pinilla C., Sette A., Peters B. (2008). Quantitative peptide binding motifs for 19 human and mouse MHC class I molecules derived using positional scanning combinatorial peptide libraries. Immunome Res..

[38] Wang P., Sidney J., Dow C., Mothé B., Sette A., Peters B. (2008). A Systematic Assessment of MHC Class II Peptide Binding Predictions and Evaluation of a Consensus Approach. PLoS Comput. Biol..

[39] Nielsen M., Lundegaard C., Lund O. (2007). Prediction of MHC class II binding affinity using SMM-align, a novel stabilization matrix alignment method. BMC Bioinform..

[40] Nielsen M., Lund O. (2009). NN-align. An artificial neural network-based alignment algorithm for MHC class II peptide binding prediction. BMC Bioinform..

[41] Sturniolo T., Bono E., Ding J., Raddrizzani L., Tuereci O., Sahin U., Braxenthaler M., Gallazzi F., Protti M.P., Sinigaglia F. (1999). Generation of tissue-specific and promiscuous HLA ligand databases using DNA microarrays and virtual HLA class II matrices. Nat. Biotechnol..

[42] Andreatta M., Karosiene E., Rasmussen M., Stryhn A., Buus S., Nielsen M. (2015). Accurate pan-specific prediction of peptide-MHC class II binding affinity with improved binding core identification. Immunogenetics.

[43] Oyarzún P., Ellis J.J., Bodén M., Kobe B. (2013). PREDIVAC: CD4+ T-cell epitope prediction for vaccine design that covers 95% of HLA class II DR protein diversity. BMC Bioinform..

[44] Jensen K.K., Andreatta M., Marcatili P., Buus S., Greenbaum J.A., Yan Z., Sette A., Peters B., Nielsen M. (2018). Improved methods for predicting peptide binding affinity to MHC class II molecules. Immunology.

[45] Calis J.J.A., Maybeno M., Greenbaum J.A., Weiskopf D., De Silva A.D., Sette A., Keşmir C., Peters B. (2013). Properties of MHC class I presented peptides that enhance immunogenicity. PLoS Comput. Biol..

[46] Gupta S., Kapoor P., Chaudhary K., Gautam A., Kumar R., Consortium O.S.D.D., Raghava G.P.S. (2013). In Silico Approach for Predicting Toxicity of Peptides and Proteins. PLoS ONE.

[47] Bui H.-H., Sidney J., Dinh K., Southwood S., Newman M.J., Sette A. (2006). Predicting population coverage of T-cell epitope-based diagnostics and vaccines. BMC Bioinform..

[48] Rice P., Longden I., Bleasby A. (2000). EMBOSS: The European Molecular Biology Open Software Suite. Trends Genet. TIG.

[49] Altschul S.F., Madden T.L., Schäffer A.A., Zhang J., Zhang Z., Miller W., Lipman D.J. (1997). Gapped BLAST and PSI-BLAST: A new generation of protein database search programs. Nucleic Acids Res..

[50] Jespersen M.C., Peters B., Nielsen M., Marcatili P. (2017). BepiPred-2.0: Improving sequence-based B-cell epitope prediction using conformational epitopes. Nucleic Acids Res..

[51] Singh H., Ansari H.R., Raghava G.P.S. (2013). Improved method for linear B-cell epitope prediction using antigen’s primary sequence. PLoS ONE.

[52] Vita R., Overton J.A., Greenbaum J.A., Ponomarenko J., Clark J.D., Cantrell J.R., Wheeler D.K., Gabbard J.L., Hix D., Sette A. (2015). The immune epitope database (IEDB) 3.0. Nucleic Acids Res..

[53] Kringelum J.V., Lundegaard C., Lund O., Nielsen M. (2012). Reliable B-cell Epitope Predictions: Impacts of Method Development and Improved Benchmarking. PLoS Comput. Biol..

[54] Sali A., Blundell T.L. (1993). Comparative protein modelling by satisfaction of spatial restraints. J. Mol. Biol..

[55] Colovos C., Yeates T.O. (1993). Verification of protein structures: Patterns of nonbonded atomic interactions. Protein Sci. Publ. Protein Soc..

[56] Wallner B., Elofsson A. (2003). Can correct protein models be identified?. Protein Sci. Publ. Protein Soc..

[57] Eisenberg D., Lüthy R., Bowie J.U. (1997). VERIFY3D: Assessment of protein models with three-dimensional profiles. Methods Enzymol..

[58] Chen V.B., Arendall W.B., Headd J.J., Keedy D.A., Immormino R.M., Kapral G.J., Murray L.W., Richardson J.S., Richardson D.C. (2010). MolProbity: All-atom structure validation for macromolecular crystallography. Acta Crystallogr. D Biol. Crystallogr..

[59] Lamiable A., Thévenet P., Rey J., Vavrusa M., Derreumaux P., Tufféry P. (2016). PEP-FOLD3: Faster de novo structure prediction for linear peptides in solution and in complex. Nucleic Acids Res..

[60] Trott O., Olson A.J. (2010). AutoDock Vina: Improving the speed and accuracy of docking with a new scoring function, efficient optimization and multithreading. J. Comput. Chem..

[61] Morris G.M., Huey R., Lindstrom W., Sanner M.F., Belew R.K., Goodsell D.S., Olson A.J. (2009). AutoDock4 and AutoDockTools4: Automated docking with selective receptor flexibility. J. Comput. Chem..

[62] Abraham M.J., Murtola T., Schulz R., Páll S., Smith J.C., Hess B., Lindahl E. (2015). GROMACS: High performance molecular simulations through multi-level parallelism from laptops to supercomputers. SoftwareX.

[63] Berendsen H.J.C., van der Spoel D., van Drunen R. (1995). GROMACS: A message-passing parallel molecular dynamics implementation. Comput. Phys. Commun..

[64] Lindorff-Larsen K., Piana S., Palmo K., Maragakis P., Klepeis J.L., Dror R.O., Shaw D.E. (2010). Improved side-chain torsion potentials for the Amber ff99SB protein force field. Proteins.

[65] Jorgensen W.L., Chandrasekhar J., Madura J.D., Impey R.W., Klein M.L. (1983). Comparison of simple potential functions for simulating liquid water. J. Chem. Phys..

[66] Verlet L. (1967). Computer “Experiments” on Classical Fluids. I. Thermodynamical Properties of Lennard-Jones Molecules. Phys. Rev..

[67] Essmann U., Perera L., Berkowitz M.L., Darden T., Lee H., Pedersen L.G. (1995). A smooth particle mesh Ewald method. J. Chem. Phys..

[68] Hess B., Bekker H., Berendsen H., Fraaije J.G.E.M. (1997). LINCS: A Linear Constraint Solver for molecular simulations. J. Comput. Chem..

[69] Bussi G., Donadio D., Parrinello M. (2007). Canonical sampling through velocity rescaling. J. Chem. Phys..

[70] Nosé S., Klein M.L. (1983). Constant pressure molecular dynamics for molecular systems. Mol. Phys..

[71] Parrinello M., Rahman A. (1981). Polymorphic transitions in single crystals: A new molecular dynamics method. J. Appl. Phys..

[72] Janeway C.A., Travers P., Walport M., Shlomchik M.J. (2001). Immunobiology.

[73] Yuliwulandari R., Kashiwase K., Nakajima H., Uddin J., Susmiarsih T.P., Sofro A.S.M., Tokunaga K. (2009). Polymorphisms of HLA genes in Western Javanese (Indonesia): Close affinities to Southeast Asian populations. Tissue Antigens.

[74] Appanna R., Ponnampalavanar S., Lum Chai See L., Sekaran S.D. (2010). Susceptible and protective HLA class 1 alleles against dengue fever and dengue hemorrhagic fever patients in a Malaysian population. PLoS ONE.

[75] Mercado E.S., Espino F.E., Perez M.L.M., Bilar J.M., Bajaro J.D.P., Huy N.T., Baello B.Q., Kikuchi M., Hirayama K. (2015). HLA-A*33:01 as Protective Allele for Severe Dengue in a Population of Filipino Children. PLoS ONE.

[76] Weiskopf D., Angelo M.A., de Azeredo E.L., Sidney J., Greenbaum J.A., Fernando A.N., Broadwater A., Kolla R.V., De Silva A.D., de Silva A.M. (2013). Comprehensive analysis of dengue virus-specific responses supports an HLA-linked protective role for CD8+ T-cells. Proc. Natl. Acad. Sci. USA.

[77] LaFleur C., Granados J., Vargas-Alarcon G., Ruíz-Morales J., Villarreal-Garza C., Higuera L., Hernández-Pacheco G., Cutiño-Moguel T., Rangel H., Figueroa R. (2002). HLA-DR antigen frequencies in Mexican patients with dengue virus infection: HLA-DR4 as a possible genetic resistance factor for dengue hemorrhagic fever. Hum. Immunol..

[78] Sierra B., Alegre R., Pérez A.B., García G., Sturn-Ramirez K., Obasanjo O., Aguirre E., Alvarez M., Rodriguez-Roche R., Valdés L. (2007). HLA-A, -B, -C, and -DRB1 allele frequencies in Cuban individuals with antecedents of dengue 2 disease: Advantages of the Cuban population for HLA studies of dengue virus infection. Hum. Immunol..

[79] Gao X., Zimmet P., Serjeantson S.W. (1992). HLA-DR,DQ sequence polymorphisms in Polynesians, Micronesians, and Javanese. Hum. Immunol..

[80] Lan N.T.P., Kikuchi M., Huong V.T.Q., Ha D.Q., Thuy T.T., Tham V.D., Tuan H.M., Tuong V.V., Nga C.T.P., Van Dat T. (2008). Protective and Enhancing HLA Alleles, HLA-DRB1*0901 and HLA-A*24, for Severe Forms of Dengue Virus Infection, Dengue Hemorrhagic Fever and Dengue Shock Syndrome. PLoS Negl. Trop. Dis..

[81] Mack S.J., Bugawan T.L., Moonsamy P.V., Erlich J.A., Trachtenberg E.A., Paik Y.K., Begovich A.B., Saha N., Beck H.P., Stoneking M. (2000). Evolution of Pacific/Asian populations inferred from HLA class II allele frequency distributions. Tissue Antigens.

[82] Shityakov S., Förster C. (2014). In silico predictive model to determine vector-mediated transport properties for the blood–brain barrier choline transporter. Adv. Appl. Bioinform. Chem. AABC.

[83] Lobanov M.Y., Bogatyreva N.S., Galzitskaya O.V. (2008). Radius of gyration as an indicator of protein structure compactness. Mol. Biol..

[84] Fan Q.R., Wiley D.C. (1999). Structure of Human Histocompatibility Leukocyte Antigen (Hla)-Cw4, a Ligand for the Kir2d Natural Killer Cell Inhibitory Receptor. J. Exp. Med..

[85] Fleischmann G., Fisette O., Thomas C., Wieneke R., Tumulka F., Schneeweiss C., Springer S., Schäfer L.V., Tampé R. (2015). Mechanistic Basis for Epitope Proofreading in the Peptide-Loading Complex. J. Immunol..

[86] Ho Z.J.M., Hapuarachchi H.C., Barkham T., Chow A., Ng L.C., Lee J.M.V., Leo Y.S., Prem K., Lim Y.H.G., de Sessions P.F. (2017). Outbreak of Zika virus infection in Singapore: an epidemiological, entomological, virological, and clinical analysis. Lancet Infect. Dis..

[87] Sant’Angelo D.B., Robinson E., Janeway C.A., Denzin L.K. (2002). Recognition of core and flanking amino acids of MHC class II-bound peptides by the T-cell receptor. Eur. J. Immunol..

[88] Quintero-Hernández V., Jiménez-Vargas J.M., Gurrola G.B., Valdivia H.H.F., Possani L.D. (2013). Scorpion venom components that affect ion-channels function. Toxicon Off. J. Int. Soc. Toxinol..

[89] Zheng J., Huang Q., Huang R., Deng F., Yue X., Yin J., Zhao W., Chen Y., Wen L., Zhou J. (2017). B-cells Are Indispensable for a Novel Mouse Model of Primary Sjögren’s Syndrome. Front. Immunol..

[90] Liu J., Gao G.F. (2011). Major Histocompatibility Complex: Interaction with Peptides. eLS.

[91] Hauser A.S., Windshügel B. (2016). LEADS-PEP: A Benchmark Data Set for Assessment of Peptide Docking Performance. J. Chem. Inf. Model..

[92] Rentzsch R., Renard B.Y. (2015). Docking small peptides remains a great challenge: An assessment using AutoDock Vina. Brief. Bioinform..

[93] Lin H.H., Zhang G.L., Tongchusak S., Reinherz E.L., Brusic V. (2008). Evaluation of MHC-II peptide binding prediction servers: Applications for vaccine research. BMC Bioinform..

[94] Lin H.H., Ray S., Tongchusak S., Reinherz E.L., Brusic V. (2008). Evaluation of MHC class I peptide binding prediction servers: Applications for vaccine research. BMC Immunol..

[95] Westermarck J., Ivaska J., Corthals G.L. (2013). Identification of Protein Interactions Involved in Cellular Signaling. Mol. Cell. Proteomics MCP.

[96] Lodish H., Berk A., Zipursky S.L., Matsudaira P., Baltimore D., Darnell J. (2000). Molecular Cell Biology.

[97] Rognan D., Scapozza L., Folkers G., Daser A. (1994). Molecular Dynamics Simulation of MHC-Peptide Complexes as a Tool for Predicting Potential T-cell Epitopes. Biochemistry.

[98] Ooi J.D., Petersen J., Tan Y.H., Huynh M., Willett Z.J., Ramarathinam S.H., Eggenhuizen P.J., Loh K.L., Watson K.A., Gan P.Y. (2017). Dominant protection from HLA-linked autoimmunity by antigen-specific regulatory T-cells. Nature.

[99] Saisawang C., Kuadkitkan A., Auewarakul P., Smith D.R., Ketterman A.J. (2018). Glutathionylation of dengue and Zika NS5 proteins affects guanylyltransferase and RNA dependent RNA polymerase activities. PLoS ONE.

[100] French S., Robson B. (1983). What is a conservative substitution?. J. Mol. Evol..

[101] Barlow D.J., Edwards M.S., Thornton J.M. (1986). Continuous and discontinuous protein antigenic determinants. Nature.

[102] Yu I.-M., Zhang W., Holdaway H.A., Li L., Kostyuchenko V.A., Chipman P.R., Kuhn R.J., Rossmann M.G., Chen J. (2008). Structure of the Immature Dengue Virus at Low pH Primes Proteolytic Maturation. Science.

[103] Zhang Y., Corver J., Chipman P.R., Zhang W., Pletnev S.V., Sedlak D., Baker T.S., Strauss J.H., Kuhn R.J., Rossmann M.G. (2003). Structures of immature flavivirus particles. EMBO J..

[104] Sirohi D., Chen Z., Sun L., Klose T., Pierson T.C., Rossmann M.G., Kuhn R.J. (2016). The 3.8 Å resolution cryo-EM structure of Zika virus. Science.

[105] Xu X., Song H., Qi J., Liu Y., Wang H., Su C., Shi Y., Gao G.F. (2016). Contribution of intertwined loop to membrane association revealed by Zika virus full-length NS1 structure. EMBO J..

[106] Jones C.T., Ma L., Burgner J.W., Groesch T.D., Post C.B., Kuhn R.J. (2003). Flavivirus Capsid Is a Dimeric Alpha-Helical Protein. J. Virol..

[107] Wen D., Li S., Dong F., Zhang Y., Lin Y., Wang J., Zou Z., Zheng A. (2018). N-glycosylation of Viral E Protein Is the Determinant for Vector Midgut Invasion by Flaviviruses. mBio.

[108] Wang Q., Yan J., Gao G.F. (2017). Monoclonal Antibodies against Zika Virus: Therapeutics and Their Implications for Vaccine Design. J. Virol..

[109] Dai L., Song J., Lu X., Deng Y.-Q., Musyoki A.M., Cheng H., Zhang Y., Yuan Y., Song H., Haywood J. (2016). Structures of the Zika Virus Envelope Protein and Its Complex with a Flavivirus Broadly Protective Antibody. Cell Host Microbe.

[110] Hasan S.S., Miller A., Sapparapu G., Fernandez E., Klose T., Long F., Fokine A., Porta J.C., Jiang W., Diamond M.S. (2017). A human antibody against Zika virus crosslinks the E protein to prevent infection. Nat. Commun..

[111] Wang Q., Yang H., Liu X., Dai L., Ma T., Qi J., Wong G., Peng R., Liu S., Li J. (2016). Molecular determinants of human neutralizing antibodies isolated from a patient infected with Zika virus. Sci. Transl. Med..

[112] Robbiani D.F., Bozzacco L., Keeffe J.R., Khouri R., Olsen P.C., Gazumyan A., Schaefer-Babajew D., Avila-Rios S., Nogueira L., Patel R. (2017). Recurrent Potent Human Neutralizing Antibodies to Zika Virus in Brazil and Mexico. Cell.

[113] Zhao H., Fernandez E., Dowd K.A., Speer S.D., Platt D.J., Gorman M.J., Govero J., Nelson C.A., Pierson T.C., Diamond M.S. (2016). Structural Basis of Zika Virus-Specific Antibody Protection. Cell.

[114] Li Y., Zhang Z., Phoo W.W., Loh Y.R., Li R., Yang H.Y., Jansson A.E., Hill J., Keller T.H., Nacro K. (2018). Structural Insights into the Inhibition of Zika Virus NS2B-NS3 Protease by a Small-Molecule Inhibitor. Structure.

[115] Badawi M.M., Osman M.M., Alla A.F., Ahmedani A.M., Abdalla M.H., Gasemelseed M.M., Elsayed A.A., Salih M.A. (2016). Highly conserved epitopes of Zika envelope glycoprotein may act as a novel peptide vaccine with high coverage: Immunoinformatics approach. Am. J. Biomed. Res..

[116] Roider J., Meissner T., Kraut F., Vollbrecht T., Stirner R., Bogner J.R., Draenert R. (2014). Comparison of experimental fine-mapping to in silico prediction results of HIV-1 epitopes reveals ongoing need for mapping experiments. Immunology.

[117] Cravo P., Machado R.B., Leite J.A., Leda T., Suwanarusk R., Bittencourt N., Albrecht L., Judice C., Lopes S.C., Lacerda M.V. (2018). In silico epitope mapping and experimental evaluation of the Merozoite Adhesive Erythrocytic Binding Protein (MAEBL) as a malaria vaccine candidate. Malar. J..

[118] Agallou M., Athanasiou E., Koutsoni O., Dotsika E., Karagouni E. (2014). Experimental validation of multi-epitope peptides including promising MHC class I-and II-restricted epitopes of four known Leishmania infantum proteins. Front. Immunol..

[119] Liljeroos L., Malito E., Ferlenghi I., Bottomley M.J. (2015). Structural and computational biology in the design of immunogenic vaccine antigens. J. Immunol. Res..

