# Supplementary material for: Peptide-Based Subunit Vaccine Design of T- and B-Cells Multi-Epitopes against Zika Virus Using Immunoinformatics Approaches
Source: Microorganisms. 2019 Jul 31;7(8):226. doi: 10.3390/microorganisms7080226 (PMC6722788; doi:10.3390/microorganisms7080226)
Supplement: Supplementary file 1 [file microorganisms-07-00226-s001.pdf]

Table S1. Zika polyprotein sequences used to obtain consensus sequence and in epitope conservancy analysis.

GenBank accession[1]	Origin	Year
ARB07967.1	Brazil	2016
ARB07962.1	Brazil	2016
AOY08541.2	Brazil	2016
AOY08538.2	Brazil	2016
AOY08518.2	Brazil	2016
AOY08517.1	Brazil	2016
ARB07981.1	Colombia	2016
ARB07978.1	Colombia	2016
ARB07988.1	Dominican Republic	2016
ARB07987.1	Dominican Republic	2016
ARB07953.1	Dominican Republic	2016
ARB07932.1	Dominican Republic	2016
ARB07927.1	Dominican Republic	2016
AOY08523.3	Dominican Republic	2016
AOY08539.3	Dominican Republic	2016
AOY08526.2	Dominican Republic	2016
AOY08542.1	Dominican Republic	2016
AOY08535.1	Dominican Republic	2016
AOY08525.1	Dominican Republic	2016
AOY08524.1	Dominican Republic	2016
AOY08521.1	Dominican Republic	2016
ARB07980.1	Florida	2016
ARB07934.1	Florida	2016
AOY08546.2	Florida	2016
AOY08537.2	Florida	2016
AOY08520.2	Florida	2016
AOY08545.1	Florida	2016
AOY08544.1	Florida	2016
AOY08543.1	Florida	2016
AOY08516.1	Florida	2016
ARB07960.1	Honduras	2016
ARB07954.1	Honduras	2016
ARB07930.1	Honduras	2016
AOY08540.2	Honduras	2016
AOY08533.2	Honduras	2016
AOY08531.2	Honduras	2016
AOY08527.2	Honduras	2016
AOY08536.1	Honduras	2016
ARB07976.1	Puerto Rico	2016
*AMD61711.1	Philippines	2012
*APH11510.1	Singapore	2016
*APH11493.1	Singapore	2016
*AMR39834.1	Cambodia	2010
*BAX00477.1	Vietnam	2016
*AMK49492.2	Indonesia	2014
*AMX81917.1	Thailand	2015
*AMD61710.1	Thailand	2014
*AMX81916.1	Thailand	2013
*AHZ13508.1	French Polynesia	2013
*ANO46301.1	French Polynesia	2013
*ANO46303.1	French Polynesia	2014
*AUI42194.1	Mexico	2016
*AUD54964.1	Mexico	2016
*ATG29267.1	Mexico	2016

[1] Sequences with * are only used in conservancy analysis.

MKNPKKKSGGFRIVNMLKRGVARVSPFGGLKRLPAGLLLGHGPIRMVLAILAFLRFTAIKPSLGLINRW
GSVGKKEAMEIIKKFKKDLAAMLRIINARKEKKRRGADTSVGIVGLLLTTAMAAEVTRRGSAYYMYLD
RNDAGEAISFPTTLGMNKCYIQIMDLGHMCDATMSYECPMLDEGVEPDDVDCWCNTTSTWVVYGTC
HHKKGEARRSRRAVTLPSHSTRKLQTRSQTWLESREYTKHLIRVENWIFRNPGFALAAAAIAWLLGSST
SQKVIYLVMILLIAPAYSIRCIGVSNRDFVEGMSGGTWVDVVLEHGGCVTVMAQDKPTVDIELVTTTVS
NMAEVRSYCYEASISDMASDSRCPTQGEAYLDKQSDTQYVCKRTLVDRGWGNGCGLFGKGSLVTCAK
FACSKKMTGKSIQPENLEYRIMLSVHGSQHSGMIVNDTGHETDENRAKVEITPNSPRAEATLGGFGSLG
LDCEPRTGLDFSDLYYLTMNNKHWLVHKEWFHDIPLPWHAGADTGTPHWNNKEALVEFKDAHAKR
QTVVVLGSQEGAVHTALAGALEAEMDGAKGRLSSGHLKCRLKMDKLRLKGVSYSLCTAAFTFTKIPAE
TLHGTVTVEVQYAGTDGPCKVPAQMAVDMQTLTPVGRLITANPVITESTENSKMMLELDPPFGDSYIVI
GVGEKKITHHWHRSGSTIGKAFEATVRGAKRMAVLGDTAWDFGSVGGALNSLGKGIHQIFGAAFKSL
FGGMSWFSQILIGTLLMWLGLNTKNGSISLMCLALGGVLIFLSTAVSADVGCSVDFSKKETRCGTGVFV
YNDVEAWRDRYKYHPDSPRRLAAAVKQAWEDGICGISSVSRMENIMWRSVEGELNAILEENGVQLTV
VVGSVKNPMWRGPQRLPVPVNELPHGWKAWGKSYFVRAAKTNNSFVVDGDTLKECPLKHRAWNSF
LVEDHGFGVFHTSVWLKVREDYSLECDPAVIGTAVKGKEAVHSDLGYWIESEKNDTWRLKRAHLIEM
KTCEWPKSHTLWTDGIEESDLIIPKSLAGPLSHHNTREGYRTQMKGPWHSEELEIRFEECPGTKVHVEET
CGTRGPSLRSTTASGRVIEEWCCRECTMPPLSFRAKDGCWYGMEIRPRKEPESNLVRSMVTAGSTDHM
DHFSLGVLVILLMVQEGLKKRMTTKIIISTSMAVLVAMILGGFSMSDLAKLAILMGATFAEMNTGGDVA
HLALIAAFKVRPALLVSFIFRANWTPRESMLLALASCLLQTAISALEGDLMVLINGFALAWLAIRAMVV
PRTDNITLAILAALTPLARGTLLVAWRAGLATCGGFMLLSLKGKGSVKKNLPFVMALGLTAVRLVDPI
NVVGLLLLTRSGKRSWPPSEVLTAVGLICALAGGFAKADIEMAGPMAAVGLLIVSYVVSGKSVDMYIER
AGDITWEKDAEVTGNSPRLDVALDESGDFSLVEDDGPPMREIILKVVLMTICGMNPIAIPFAAGAWYVY
VKTGKRSGALWDVPAPKEVKKGETTDGVYRVMTRRLLGSTQVGVGVMQEGVFHTMWHVTKGSALR
SGEGRLDPYWGDVKQDLVSYCGPWKLDAAWDGHSEVQLLAVPPGERARNIQTLPGIFKTKDGDIGAV
ALDYPAGTSGSPILDKCGRVIGLYGNGVVIKNGSYVSAITQGRREEETPVECFEPSMLKKKQLTVLDLHP
GAGKTRRVLPEIVREAIKTRLRTVILAPTRVVAAEMEEALRGLPVRYMTTAVNVTHSGTEIVDLMCHAT
FTSRLLQPIRVPNYNLYIMDEAHFTDPSSIAARGYISTRVEMGEAAAIFMTATPPGTRDAFPDSNSPIMDT
EVEVPERAWSSGFDWVTDHSGKTVWFVPSVRNGNEIAACLTKAGKRVIQLSRKTFETEFQKTKHQEW
DFVVTTDISEMGANFKADRVIDSRRCLKPVILDGERVILAGPMPVTHASAAQRRGRIGRNPNKPGDEYL
YGGGCAETDEDHAHWLEARMLLDNIYLQDGLIASLYRPEADKVAAIEGEFKLRTEQRKTFVELMKRG
DLPVWLAYQVASAGITYTDRRWCFDGTTNNTIMEDSVPAEVWTRHGEKRVLKPRWMDARVCSDHA
ALKSFKEFAAGKRGAAFGVMEALGTLPGHMTERFQEAIDNLAVLMRAETGSRPYKAAAAQLPETLETI
MLLGLLGTVSLGIFFVLMRNKGIGKMGFGMVTLGASAWLMWLSEIEPARIACVLIVVFLLLVVLIPEPEK
QRSPQDNQMAIIIMVAVGLLGLITANELGWLERTKSDLSHLMGRREEGATIGFSMDIDLRPASAWAIYA
ALTTFITPAVQHAVTTSYNNYSLMAMATQAGVLFGMGKGMPFYAWDFGVPLLMIGCYSQLTPLTLIV
AIILLVAHYMYLIPGLQAAAARAAQKRTAAGIMKNPVVDGIVVTDIDTMTIDPQVEKKMGQVLLIAVA
VSSAILSRTAWGWGEAGALITAATSTLWEGSPNKYWNSSTATSLCNIFRGSYLAGASLIYTVTRNAGLV
KRRGGGTGETLGEKWKARLNQMSALEFYSYKKSGITEVCREEARRALKDGVATGGHAVSRGSAKLRW
LVERGYLQPYGKVIDLGCGRGGWSYYAATIRKVQEVKGYTKGGPGHEEPVLVQSYGWNIVRLKSGVD
VFHMAAEPCDTLLCDIGESSSSPEVEEARTLRVLSMVGDWLEKRPGAFCIKVLCPYTSTMMETLERLQR
RYGGGLVRVPLSRNSTHEMYWVSGAKSNTIKSVSTTSQLLLGRMDGPRRPVKYEEDVNLGSGTRAVVS
CAEAPNMKIIGNRIERIRSEHAETWFFDENHPYRTWAYHGSYEAPTQGSASSLINGVVRLLSKPWDVVT
GVTGIAMTDTTPYGQQRVFKEKVDTRVPDPQEGTRQVMSMVSSWLWKELGKHKRPRVCTKEEFINKV
RSNAALGAIFEEEKEWKTAVEAVNDPRFWALVDKEREHHLRGECQSCVYNMMGKREKKQGEFGKAK
GSRAIWYMWLGARFLEFEALGFLNEDHWMGRENSGGGVEGLGLQRLGYVLEEMSRIPGGRMYADDT
AGWDTRISRFDLENEALITNQMEKGHRALALAIIKYTYQNKVVKVLRPAEKGKTVMDIISRQDQRGSG
QVVTYALNTFTNLVVQLIRNMEAEEVLEMQDLWLLRRSEKVTNWLQSNGWDRLKRMAVSGDDCVV
KPIDDRFAHALRFLNDMGKVRKDTQEWKPSTGWDNWEEVPFCSHHFNKLHLKDGRSIVVPCRHQDE
LIGRARVSPGAGWSIRETACLAKSYAQMWQLLYFHRRDLRLMANAICSSVPVDWVPTGRTTWSIHGK
GEWMTTEDMLVVWNRVWIEENDHMEDKTPVTKWTDIPYLGKREDLWCGSLIGHRPRTTWAENIKNT
VNMVRRIIGDEEKYMDYLSTQVRYLGEEGSTPGVL.

Diagram S1. Consensus Zika polyprotein sequence

Table S2. Validation of homology modeled MHC structures and their template structures.

Molecule	Structure	Errat[1]		ProQ[2]		Verify3D[3]	MolProbity[4]						
		QF A	QF B	LGscore	MaxSub	3D/1D profile >= 0.2	Poor rotamers	Favored rotamers	Ramachandran outliers	Ramachandran favored	Cβ deviations >0.25Å	Bad bonds	Bad angles
DQA1*0501/DQB1*0303	Template 1UVQ	93.49	92.12	4.774	0.361	78.07%	0.00%	99.11%	0.27%	97.33%	0.00%	0.00%	0.02%
	Model	83.62	79.55	4.059	0.291	70.00%	2.06%	96.18%	0.82%	96.99%	1.13%	0.06%	1.30%
DQA1*0201/DQB1*0303	Template 1UVQ	93.49	92.12	4.774	0.361	78.07%	0.00%	99.11%	0.27%	97.33%	0.00%	0.00%	0.02%
	Model	72.09	79.01	4.118	0.304	74.93%	1.75%	93.57%	1.09%	96.46%	0.84%	0.06%	1.38%
DRB1*1101	Template 4MD5	78.95	96.05	3.975	0.331	78.53%	1.08%	95.95%	0.00%	98.40%	0.00%	0.09%	0.02%
	Model	59.88	89.01	3.749	0.302	79.46%	0.89%	96.75%	0.00%	98.09%	0.28%	0.00%	1.16%
DRB1*0901	Template 5V4M	78.24	90.81	4.68	0.371	85.45%	0.87%	93.62%	0.27%	98.66%	0.00%	0.03%	0.02%
	Model	58.82	77.90	4.168	0.315	82.02%	0.60%	95.20%	0.00%	98.07%	0.29%	0.00%	1.28%

[1] QF: Quality Factor, for chains A and B [97].

[2] LGscore: >1.5 = fairly good; >2.5 = very good; > 4 = extremely good [98]. MaxSub: >0.1 = fairly good; >0.5 = very good; >0.8 = extremely good [98]

[3] Verify3D target for 3D/1D profile >= 0.2 is >80% [99].

[4] MolProbity targets for: Poor rotamer: <0.3%; Favored rotamer: >98%; Ramachandran outliers: <0.05%; Ramachandran favored: >98%; Cβ deviations >0.25A: 0; Bad bonds: 0%; Bad angles: <0.1% [100].

Table S3. List of hydrogen bonds for docked MHC-I epitopes.

MVLAILAFL			YMWLGARFL		
Ligand	Receptor	Distance (Å)	Ligand	Receptor	Distance (Å)
Met1:N	Tyr171:OH	2.71	Tyr1:N	Tyr171:OH	2.77
Met1:N	Tyr7:OH	2.87	Tyr1:N	Tyr7:OH	2.99
Met1:O	Tyr159:OH	2.7	Tyr1:O	Tyr159:OH	3.27
Val2:N	Glu63:OE1	2.99	Met2:N	Glu63:OE2	3.03
Val2:O	Lys66:NZ	3.07	Leu4:O	Arg97:NH2	2.79
Leu3:N	Tyr99:OH	2.92	Arg7:NH1	Tyr116:OH	3.13
Ala7:O	Tyr116:OH	2.74	Arg7:NH2	Tyr116:OH	2.78
Phe8:O	Trp147:NE1	2.91	Arg7:O	Trp147:NE1	3.01
Leu9:N	Asp77:OD1	3.1			

YAWDFGVPL			RYMTTAVNV		
Ligand	Receptor	Distance (Å)	Ligand	Receptor	Distance (Å)
Tyr1:N	Tyr171:OH	2.8	Arg1:NH2	Glu63:OE2	3.33
Tyr1:O	Tyr159:OH	2.68	Arg1:NE	Glu63:OE2	2.8
Tyr1:N	Tyr7:OH	3.05	Arg1:N	Tyr171:OH	3.07
Ala2:O	Lys66:NZ	3.22	Arg1:N	Tyr7:OH	2.86
Trp3:N	Tyr99:OH	3.09	Arg1:O	Tyr159:OH	2.69
Pro8:O	Trp147:NE1	2.8	Asn8:ND2	Thr80:OG1	3.15
Leu9:N	Asp77:OD1	2.97	Asn8:O	Trp147:NE1	2.85
			Asn8:ND2	Asp77:OD1	2.86
			Val9:N	Asp77:OD1	2.8

WYMWLGARF			AIYAALTTF		
Ligand	Receptor	Distance (Å)	Ligand	Receptor	Distance (Å)
Tyr2:OH	His70:ND1	3.04	Ala1:N	Tyr171:OH	3.28
Trp4:NE1	Tyr99:OH	3.05	Ala1:N	Tyr59:OH	3.19
Arg8:NE	Thr73:OG1	3.11	Ala1:N	Tyr7:OH	3.12
Arg8:NH2	Thr73:OG1	3	Ala1:O	Tyr159:OH	2.55
Arg8:NH1	Gln72:OE1	3.1	Ile2:N	Glu63:OE1	2.94
Arg8:NH2	Gln72:OE1	3.12	Ile2:O	Arg62:NH1	3.03
Arg8:O	Phe9:NZ	2.92	Ile2:O	Arg62:NH2	2.99
			Tyr3:N	Tyr99:OH	2.79
			Thr7:OG1	Asn70:OD1	2.77
			Thr7:OG1	Thr73:OG1	2.98
			Thr8:O	Trp147:NE1	2.9
			Phe9:N	Ser77:OG	3.1

FAAGAWYVY			IAMTDTTPY		
Ligand	Receptor	Distance (Å)	Ligand	Receptor	Distance (Å)
Phe1:N	Asn63:OD1	3.27	Ile1:N	Tyr59:OH	2.82
Phe1:N	Tyr59:OH	2.85	Ile1:N	Asn63:OD1	3.16
Phe1:N	Tyr171:OH	3.3	Ile1:N	Tyr7:OH	2.81
Phe1:N	Tyr7:OH	2.8	Ala2:N	Tyr7:OH	3.13
Ala2:O	Tyr159:OH	3.06	Ala2:O	Tyr159:OH	2.85
Tyr7:O	Trp147:NE1	3.06	Pro8:O	Trp147:NE1	3.08
Val8:O	Trp147:NE1	3.17	Tyr9:N	Ser77:OG	3.02
Tyr9:N	Ser77:OG	3.02	Tyr9:OH	Ser116:OG	2.8
Tyr9:OH	Ser116:OG	2.81			

WFHDIPLPW		
Ligand	Receptor	Distance (Å)
Trp1:N	Tyr7:OH	2.91

Trp1:N	Tyr171:OH	2.95
Trp1:O	Tyr159:OH	2.65
Phe2:N	Glu63:OE2	2.87
Asp4:OD2	Gln70:NE2	3.16
Ile5:O	Arg156:NH2	3.33
Leu7:N	Gln70:OE1	3.06
Pro8:O	Trp147:NE1	2.8

Table S4. List of hydrogen bonds for docked MHC-II epitopes.

**IAVAVSSAI**			**IEMAGPMAA**		
Ligand	Receptor	Distance (Å)	Ligand	Receptor	Distance (Å)
Ala2:N	A:Tyr11:OH	2.89	Ile1:N	B:Asp57:OD1	2.81
Ser6:OG	A:Thr63:OG	2.92	Glu2:OE2	A:Asn71:ND2	3.16
Ser7:O	B:Arg70:NH1	3.08	Glu2:OE1	B:Arg70:NH2	3.08
Ser7:O	B:Arg70:NH2	3.05	Glu2:O	B:Trp61:NE1	3.24
**SLMAMATQA**			**YRIMLSVHG**		
Ligand	Receptor	Distance (Å)	Ligand	Receptor	Distance (Å)
Ser1:OG	A:Leu54:OG	3.02	Tyr1:N	A:Ser53:O	3.2
Ser1:N	A:Arg55:O	3.08	Arg2:N	B:Asn82:OD1	2.93
Ala4:O	B:Thr77:OG1	2.84	Arg2:O	B:Asn82:ND2	3.16
Met5:O	B:Arg70:NH2	2.77	Met4:N	A:Gln9:OE1	3
Ala6:N	B:Glu74:OE2	2.78	Met4:O	A:Gln9:NE2	3.14
Gln8:NE2	B:Tyr47:OH	2.99	Val7:N	B:Tyr30:OH	2.91
			Val7:O	A:Asn69:ND2	3.29
			His8:O	B:Trp61:NE1	3.11
			Gly9:N	A:Asn69:OD1	2.99
**LYFHRRDLR**			**IILLVAHYM**		
Ligand	Receptor	Distance (Å)	Ligand	Receptor	Distance (Å)
Tyr2:OH	A:Ser53:OH	2.87	Ile1:N	A:Ser53:O	3.11
His4:ND1	A:Asn62:OD1	3.01	Leu4:O	A:Gln9:NE2	2.82
Arg5:O	A:Asn62:ND2	3.1	Leu4:O	A:Asn62:ND2	3.2
Arg6:NF	A:Glu11:OE2	2.94	Val5:O	B:Gln70:NE2	2.78
Arg6:NH2	A:Glu11:OE2	2.92	Ala6:O	B:Tyr30:OH	2.84
Arg6:NH2	B:Ser13:OG	2.98	His7:NE2	A:Asp66:OD1	3.32
Arg6:O	B:Arg74:NH2	3.19			
Asp7:O	A:Asn69:ND2	3.11			
Leu8:O	B:Trp61:NE1	2.93			
Arg9:N	A:Asn69:OD1	3.04			
Arg9:NH1	B:Asp57:OD2	2.84			
Arg9:NH2	B:Glu9:OE2	2.74			
**FKKDLAAML**			**WAIYAALTT**		
Ligand	Receptor	Distance (Å)	Ligand	Receptor	Distance (Å)
Lys3:NZ	A:Glu55:O	3.21	Trp1:N	A:Ser53:O	2.8
Lys2:NZ	A:Glu55:OE1	3.22	Ala2:N	B:Asn82:OD1	3.01
Asp4:O	A:Gln9:NE2	3.17	Ala2:O	B:Asn82:ND2	2.92
Asp4:O	A:Asn62:ND2	2.85	Tyr4:N	A:Gln9:OE1	3.09
Leu5:O	B:His28:NE2	3.03	Ala5:O	A:Asn62:ND2	3
			Leu7:O	A:Asn69:ND2	2.87
			Thr9:N	A:Asn69:OD1	2.88

Table S5. List of consensus linear B cell epitopes, scores averaged across all residues, and their filtration results.

Prot	Pos	Len	Peptide	BepiPred mean	LBtope mean	Toxic[1]	Conservancy[1]	Antigenic[1]	Autoimmune[1]
C	24	6	VSPFGG	54.69	66.02	O	O	O	X
	68	10	RWGSVGKKEA	55.70	62.75	O	O	X	O
prM	56	10	LDEGVEPDDV	55.92	68.85	O	O	O	O
	84	20	KKGEARRSRRAVTLPSHSTR	56.74	79.96	O	O	O	O
M	25	7	YTKHLIR	52.44	67.70	O	O	O	O
E	89	15	QYVCKRTLVDRGWGN	55.04	79.01	O	O	O	O
	126	9	TGKSIQPEN	51.17	81.88	O	O	X	O
	146	8	SQHSGMIV	61.67	67.73	O	O	O	O
	216	7	EWFHDIP	53.29	65.21	O	O	O	O
	396	12	ITHHWHRSGSTI	51.95	67.45	O	O	X	O
	428	9	AWDFGSVGG	56.27	63.03	O	O	O	O
NS1	28	13	WRDRYKYHPDSPR	55.71	84.05	O	O	O	O
	94	8	KNPMWRGP	59.70	64.42	O	X	X	O
	141	8	KECPLKHR	54.71	64.05	O	O	O	O
	253	12	HHNTREGYRTQM	63.38	69.13	O	X	O	O
	293	11	TRGPSLRSTTA	56.56	64.73	O	O	X	O
	338	11	RKEPESNLVRS	54.47	74.24	O	O	O	O
NS3	6	7	DVPAPKE	52.23	67.54	O	O	O	O
	27	7	TRRLLGS	54.38	73.89	O	O	O	O
	58	6	LRSGEG	50.84	66.41	O	O	X	X
	65	11	LDPYWGDVKQD	53.12	69.23	O	O	O	O
	82	9	PWKLDAAWD	54.20	74.98	O	O	O	O
	110	6	QTLPGI	51.56	69.35	O	X	O	X
	328	21	DSNSPIMDTEVEVPERAWSSG	59.59	68.99	O	X	X	O
	479	9	ETDEDHAHW	55.06	69.22	O	O	X	O
	557	24	TDRRWCFDGTTNNTIMEDSVPAEV	56.55	68.56	O	X	O	O
	594	11	WMDARVCSDHA	54.89	68.68	O	O	O	O
NS4B	207	7	WEGSPNK	52.24	67.88	O	O	X	O
NS5	34	15	EVCREEARRALKDGV	52.57	76.82	O	O	O	O
	153	9	SPEVEEART	57.22	81.69	O	O	O	O
	196	7	ERLQRRY	52.51	71.37	O	X	X	O
	247	17	PRRPVKYEEDVNLGSGT	55.39	76.29	O	O	O	O
	295	7	FDENHPY	53.20	70.58	O	X	X	O
	353	9	QRVFKEKVD	56.86	63.41	O	O	O	O
	363	7	RVPDPQE	60.04	64.55	O	O	O	O
	414	24	FEEEKEWKTAVEAVNDPRFWALVD	51.84	71.12	O	O	O	O
	466	6	FGKAKG	57.25	68.95	X	O	X	X
	598	9	QDQRGSGQV	58.31	76.80	O	O	O	O
	696	10	WKPSTGWDNW	59.32	68.56	O	X	X	O
	822	9	ENDHMEDKT	57.09	70.84	O	O	X	O
	840	16	YLGKREDLWCGSLIGH	55.43	64.29	O	X	O	O
	857	7	PRTTWAE	54.88	71.57	O	O	X	O

[1] X = eliminated (toxic/nonconserved/nonantigenic/autoimmune risk), O = not eliminated.

Table S6. Validation of homology modeled MHC structures and their template structures.

Molecule	Structure	Errat[1]	ProQ[2]		Verify3D[3]	MolProbity[4]						
		QF	LGscore	MaxSub	3D/1D profile >= 0.2	Poor rotamers	Favored rotamers	Ramachandran outliers	Ramachandran favored	Cβ deviations >0.25Å	Bad bonds	Bad angles
prM	Template 4B03	NA	9.558	1.099	NA	NA	NA	NA	NA	NA	NA	NA
	Model	16.89	1.811	0.212	39.29%	2.78%	90.28%	3.61%	88.55%	0.00%	0.00%	1.24%
NS3 Protease	Template 5YOF	98.56	4.611	0.363	89.74%	0.85%	95.73%	0.00%	97.40%	0.00%	0.00%	0.00%
	Model	86.39	4.945	0.387	89.74%	0.81%	97.56%	0.00%	100.00%	0.00%	0.00%	0.61%
NS3 Helicase	Template 5VI7	95.62	6.396	0.292	92.87%	0.54%	95.92%	0.23%	97.22%	0.00%	0.00%	0.00%
	Model	82.22	6.037	0.53	86.85%	1.34%	95.43%	0.91%	97.04%	0.48%	0.03%	1.14%
NS5	Template 5U0B	94.59	5.608	0.455	91.85%	5.84%	85.13%	0.34%	94.67%	0.00%	0.00%	0.00%
	Model	83.2	5.56	0.45	90.83%	2.25%	94.30%	0.00%	97.62%	0.37%	0.00%	1.15%

[1] QF: Quality Factor, for chains A and B [97].

[2] LGscore: >1.5 = fairly good; >2.5 = very good; > 4 = extremely good [98]. MaxSub: >0.1 = fairly good; >0.5 = very good; >0.8 = extremely good [98]

[3] Verify3D target for 3D/1D profile >= 0.2 is >80% [99].

[4] MolProbity targets for: Poor rotamer: <0.3%; Favored rotamer: >98%; Ramachandran outliers: <0.05%; Ramachandran favored: >98%; Cβ deviations >0.25A: 0; Bad bonds: 0%; Bad angles: <0.1% [100].
